# Photocrosslinking Probes Proximity of Thymine Modifiers Tethering Excitonically Coupled Dye Aggregates to DNA Holliday Junction

**DOI:** 10.3390/molecules27134006

**Published:** 2022-06-22

**Authors:** Shibani Basu, Keitel Cervantes-Salguero, Bernard Yurke, William B. Knowlton, Jeunghoon Lee, Olga A. Mass

**Affiliations:** 1 Micron School of Materials Science & Engineering, Boise State University, Boise, ID 83725, USA; shibanibasu@boisestate.edu (S.B.); keitelcervantess@boisestate.edu (K.C.-S.); bernardyurke@boisestate.edu (B.Y.); bknowlton@boisestate.edu (W.B.K.); 2 Department of Electrical & Computer Engineering, Boise State University, Boise, ID 83725, USA; 3 Department of Chemistry and Biochemistry, Boise State University, Boise, ID 83725, USA

**Keywords:** exciton, squaraine, Kasha, excitonic coupling, quantum computing, dimer, tetramer

## Abstract

A DNA Holliday junction (HJ) has been used as a versatile scaffold to create a variety of covalently templated molecular dye aggregates exhibiting strong excitonic coupling. In these dye-DNA constructs, one way to attach dyes to DNA is to tether them via single long linkers to thymine modifiers incorporated in the core of the HJ. Here, using photoinduced [2 + 2] cycloaddition (photocrosslinking) between thymines, we investigated the relative positions of squaraine-labeled thymine modifiers in the core of the HJ, and whether the proximity of thymine modifiers correlated with the excitonic coupling strength in squaraine dimers. Photocrosslinking between squaraine-labeled thymine modifiers was carried out in two distinct types of configurations: adjacent dimer and transverse dimer. The outcomes of the reactions in terms of relative photocrosslinking yields were evaluated by denaturing polyacrylamide electrophoresis. We found that for photocrosslinking to occur at a high yield, a synergetic combination of three parameters was necessary: adjacent dimer configuration, strong attractive dye–dye interactions that led to excitonic coupling, and an A-T neighboring base pair. The insight into the proximity of dye-labeled thymines in adjacent and transverse configurations correlated with the strength of excitonic coupling in the corresponding dimers. To demonstrate a utility of photocrosslinking, we created a squaraine tetramer templated by a doubly crosslinked HJ with increased thermal stability. These findings provide guidance for the design of HJ-templated dye aggregates exhibiting strong excitonic coupling for exciton-based applications such as organic optoelectronics and quantum computing.

## 1. Introduction

Deoxyribonucleic acid (DNA) has been broadly utilized as a template to create excitonically coupled molecular dye aggregates [1,2,3,4,5,6,7,8,9,10,11,12,13,14,15,16,17,18,19,20,21,22,23,24,25]. In a molecular dye aggregate, in which two or more dyes are closely positioned, the excited state of one dye can be shared with a neighboring dye in the form of a delocalized Frenkel exciton [26]. According to Kasha’s molecular exciton model, the strength of excitonic coupling can be quantitatively described with an exciton hopping parameter, *J_m,n_* [26]. The creation of excitons with strong excitonic coupling is the foundation of the development of such applications as organic photoswitches [27,28,29,30,31,32], light harvesting [33], and emerging quantum information systems [34,35]. Control over intermolecular distances between dyes and their respective orientation, as well as the number of dyes per aggregate, is key in harnessing molecular excitons. Covalent tethering of dyes to the specific sites of DNA backbone or nucleobases has proved to be an effective way to create dye aggregates with strong excitonic coupling [1,2,3,4,5,6,7,8,9,10,11,12,13,14,15,16,17,18,19,20,21].

In addition to the work of others [1,2,3,4,5,7,8,10,15,36,37], our group has utilized DNA templates to create excitonically coupled aggregates of polymethine dyes such as Cy5 [38,39,40,41,42,43]. In addition, we have used a four-arm DNA nanostructure known as the DNA Holliday junction (HJ) [44,45] as a superior template, compared with simple DNA duplexes [38,39,40,41,46,47,48]. The HJ allowed us to form aggregates beyond two dyes (a dimer), expanding to three or four dyes (trimers and tetramers, respectively). While we discovered the utility of the HJ in its mobile structure characterized by sequence symmetry [39,40], we further proceeded with employing an immobile HJ with asymmetric sequences to prevent junction sliding. By covalently tethering dyes to the immobile HJ, we created dimer, trimer, and tetramer aggregates of Cy5 [38,41,42], dimers and tetramers of squaraines [47,48], and dimers of squaraine–rotaxanes [46] exhibiting excitonic coupling from a medium to strong coupling regime.

In the core of an HJ, a dye dimer can be attached to DNA in six configurations of attachment sites: two configurations in transverse fashion with dyes attached to non-complementary strands (further referred to as a transverse dimer), and four configurations in adjacent fashion with dyes attached to partially complementary strands (further referred to as an adjacent dimer). These dimer configurations were created using three types of linkers—a phosphoramidite linker [38,39,40,41], an abasic unilinker [48], and a thymine-modifier linker [47]. With the former linker, both ends of the dye are tethered to DNA, while with the latter two linkers, one end of the dye is tethered to a DNA backbone or a thymine nucleobase via a flexible single carbon chain. Our previous findings showed that spectral properties, excitonic coupling strength, and dye dimer geometry varied with a configuration of dye dimer attachment for the same dye [38,47,48]. A notable example is a dichloroindolenine squaraine dimer tethered to the HJ via thymine modifiers. The dimer adopted distinct geometries in the adjacent and in transverse configurations resulting, respectively, in *J_m,n_* of 132 meV, and 80 meV, as was determined by modeling of experimental spectral data using the Kühn–Renger–May-based approach (KRM) [47]. This difference in the excitonic coupling strengths between two dimer configurations of the same dye stems from the difference in the intermolecular dye distances and dimer geometries, which, in turn, presumably originates from the distance between dye-labeled thymine modifiers in the HJ. However, due to construct complexity, characterization methods such as X-ray crystallography or nuclear magnetic resonance are not yet feasible to elucidate the intermolecular distance between dye-labeled thymines in the HJ covalently tethering dye aggregates.

**Figure 1:**
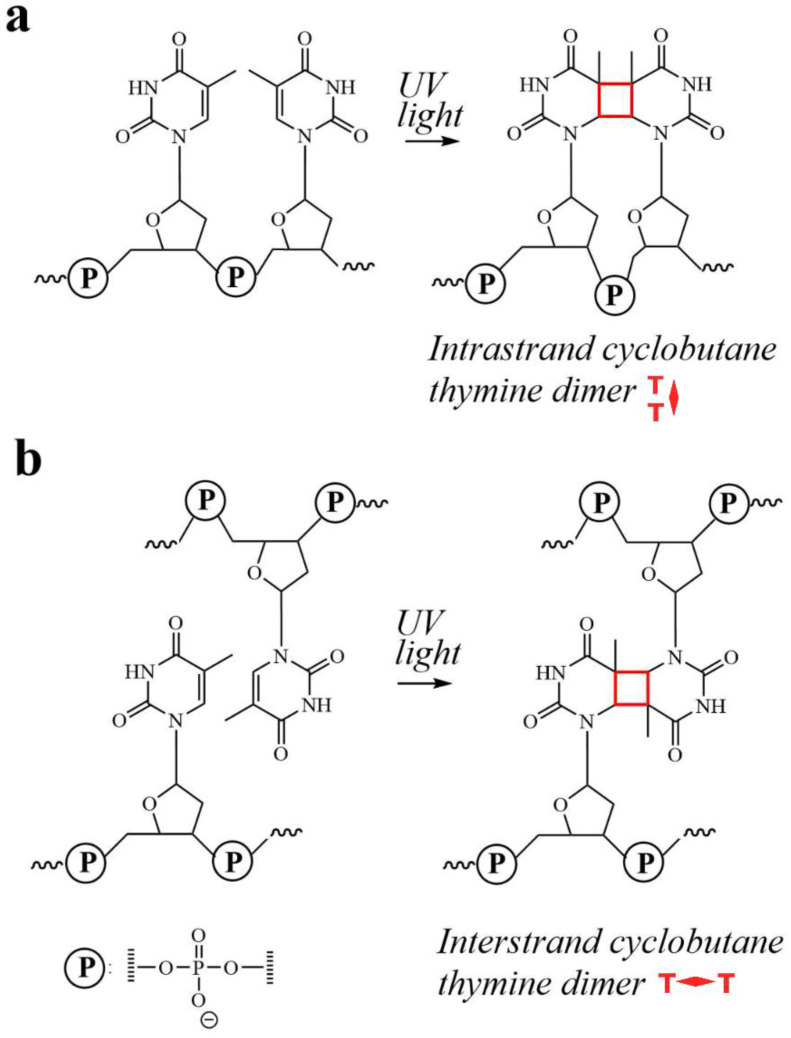
Photoinduced formation of a crosslink between thymines on DNA. (**a**) An intrastrand crosslink between adjacent thymines. (**b**) An interstrand crosslink between opposing thymines.

Here, we employed photocrosslinking of thymine-modifiers as a chemical tool to probe the proximity of dye-modified thymines tethering dye aggregates to HJs. The thymine photocrosslinking is known to proceedas a [2 + 2] photocycloaddition reaction to afford an adduct of two thymines bridged via a cyclobutane ring [49]. This reaction requires a specific stack-wise geometric alignment, i.e., conformation, of thymines to allow the π-systems of the reacting double bonds to approach each other at slightly above 3 Å [50]. When adjacent thymines are on the same strand, such favorable thymine conformation is readily achievable, leading to intrastrand crosslinks in natural and synthetic DNA constructs upon irradiation with UV light (Figure 1a) [51,52]. In contrast, photocrosslinking of thymines on opposing strands (Figure 1b), in general, does not occur under aqueous conditions as the double stranded DNA scaffold inhibites the stacked-wise proximity of the thymines. Only a few examples of photoinduced interstrand crosslinks were reported in higher order DNA structures where DNA folding forced proximate alignment of thymines of opposing strands [53,54]. Motivated by these reports, we hypothesized that photocrosslinking of dye-labeled thymine modifiers located on opposing strands in the core of the HJ could occur in some dye dimer configurations, and thus provide insight into the relative positions of dye-labeled thymines in the HJs and the role of their proximity on the excitonic coupling strength in dye aggregates.

In this work, we examined photocrosslinking between two opposing thymine modifiers covalently tethering squaraine dimers to HJs. In particular, we tested two types of dye-labeled thymine placements in HJ—the transverse dimer with dye-labeled thymine modifiers on non-complementary strands (two configurations) and the adjacent dimer with dye-labeled thymine modifiers on partially complementary strands (four configurations). The reaction outcomes were evaluated in terms of relative yield of photocrosslinking using band quantification of electrophoretic gel images. We found that photocrosslinking did not occur in the unmodified HJ, but occurred to a small extent in HJs with two unlabeled thymine modifiers or with one dye-labeled thymine modifier. Additionally, in all but one dimer configuration, photocrosslinking proceeded only to modest degrees. One adjacent dimer configuration of dichloroindolenine squaraine dimer afforded a significantly higher photocrosslinking yield (45.1%) compared with all other tested constructs. Additional experiments elucidated the role of the neighboring base pair on the photocrosslinking yield. We found that a high yield of photocrosslinking could be achieved by a synergetic combination of the placement of thymine modifiers on partially complementary strands in the HJ (adjacent dimer configuration), strongly attractive dye–dye interactions, and the A-T neighboring base pair. Altogether, the photocrosslinking method indicated that in the transverse configuration, dye-labeled thymines were rather distant most of the time. Conversely, placement in the adjacent configuration provided closer positioning of thymine-modifiers, and their proximity was further promoted by dye–dye interactions. The proximity of thymine modifiers in adjacent versus transverse dimers correlated with the excitonic coupling strength previously quantified for these dimers by KRM [47]. An important implication of these results was that photocrosslinking of dye-labeled thymines could be employed to stabilize HJ-templated dye aggregates as well as higher order DNA structures with the HJ motifs. As an example, we showed here that using this method, squaraine dimers templated by photocrosslinked HJs could be reconstructed into a squaraine tetramer templated by a doubly crosslinked HJ with increased stability as assessed by thermal denaturation. These findings open opportunities for the design of superior dye-DNA architectures for science and biological applications.

## 2. Results and Discussion

### 2.1. Molecular Design

**Figure 2:**
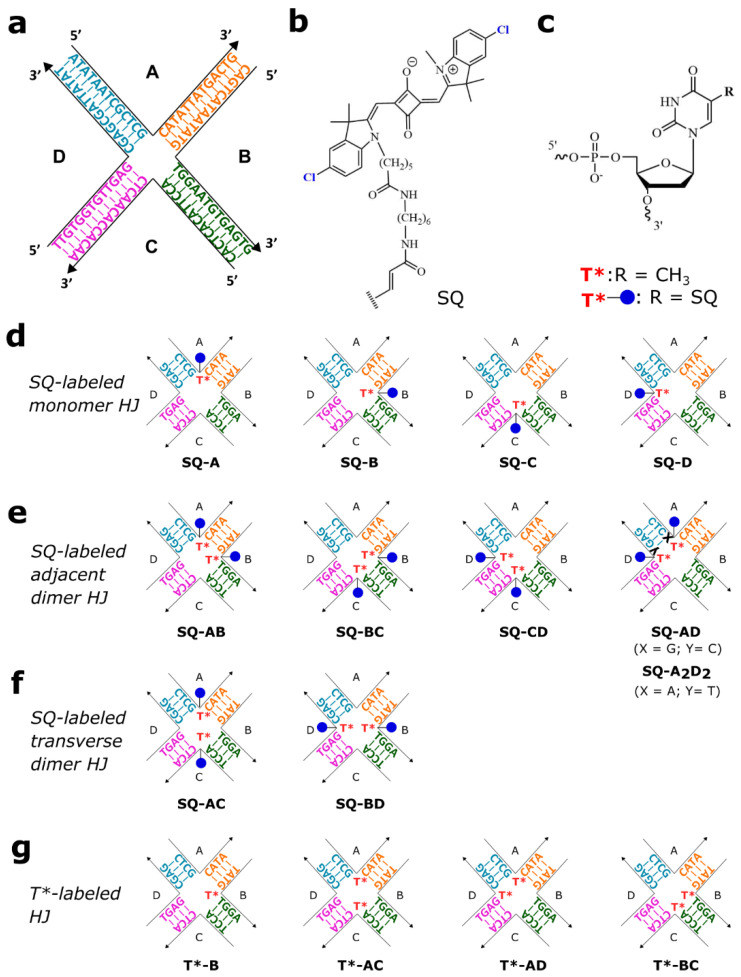
Structures of dye-DNA constructs. (**a**) Base sequence of the unmodified four-arm immobile DNA Holliday junction. The constituent single strands of DNA HJ are labeled A, B, C, and D; the complementary regions are color-coded. (**b**) Chemical structure of dichloroindolenine squaraine and tether. (**c**) Chemical structure of the unlabeled thymine modifier (T*) and thymine-modifier tethered to the squaraine (T*-blue circle). (**d**–**g**) The central sequences of the modified HJs depicting the positions of squaraine-labeled and unlabeled thymine modifiers T*. Blue circles depict thymine modifiers tethering a squaraine dye. The base sequences beyond the central four base pairs of each arm are identical to those in the unmodified HJ.

Our group employed a four-arm immobile DNA HJ (Figure 2a) as a template to create dye aggregates and to study their optical properties with a focus on the exciton coupling strength that enables a delocalized exciton [38,46,47,48]. Following our prior study that revealed that dichloroindolenine squaraine dyes form HJ-templated aggregates with exceptionally strong excitonic coupling [47], in this study we use the same DNA HJ and the method for squaraine attachment to DNA (Section S1). In particular, dichloroindolenine squaraines carrying an *N*-pentyl-NHS ester were subjected to esterification with the amino C6 thymine modifier incorporated in the center of a single strand between 13 nucleotide-long domains to afford a squaraine tethered to DNA via a single linker as shown in Figure 2b,c. Using the squaraine-labeled strands and unlabeled strands, four monomers (Figure 2d) and two types of squaraine dimers covalently templated by HJ were prepared (Figure 2e,f). The first type of dimer was an adjacent dimer, where squaraines were tethered to thymine modifiers on two partially complementary strands to afford four configurations of adjacent dimers (**SQ-AB**, **SQ-AD**, **SQ-BC**, and **SQ-CD**). The second type of dimer type was a transverse dimer in two configurations where the squaraine-labeled thymine modifiers were positioned at the non-complementary strands A and C (**SQ-AC** dimer) or B and D (**SQ-BD** dimer). Dye-labeled thymine modifiers as unpaired thymines did not participate in forming Watson–Crick base pairs but rather were thought to serve as reaction sites for photocrosslinking upon UV irradiation. 

Two types of control constructs were prepared to evaluate photocrosslinking between squaraine-labeled thymine modifiers in the HJ core. The first control was an unmodified HJ prepared by hybridization of four single stranded DNA (ssDNA) to afford an immobile 4-arm HJ where each arm was a 13-mer dsDNA (Figure 2a). The second type of control were HJs with one or two unlabeled thymine modifiers placed in the center of a strand. Thus, in the construct T*-B, one unlabeled thymine modifier was placed in strand B. In transverse T*-AC construct, two unlabeled thymine modifiers were positioned on non-complementary strands A and C, and in the adjacent T*-BC and T*-AD constructs, thymine modifiers were positioned on two partially complementary strands B and C, or A and D, respectively (Figure 2g).

### 2.2. Photocrosslinking in HJ Constructs

**Figure 3:**
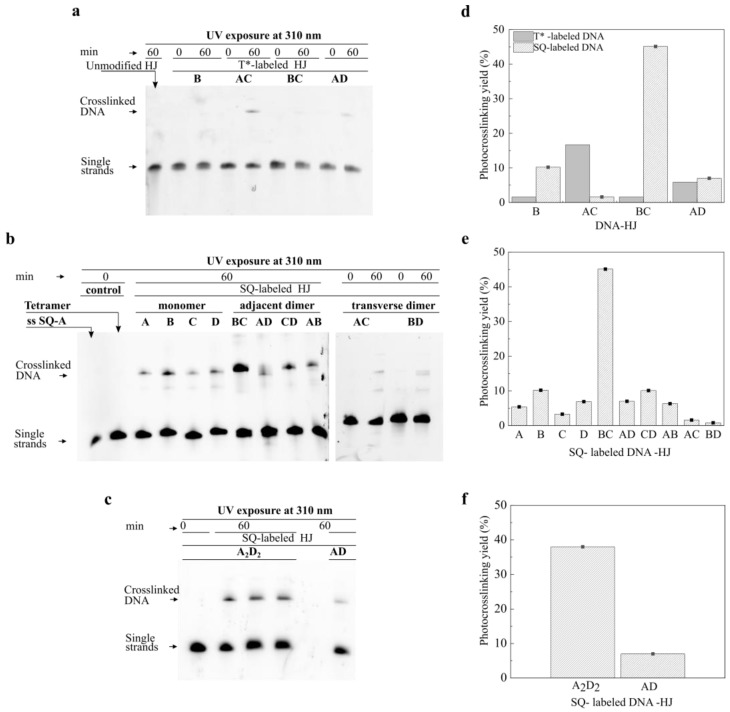
Electrophoretic analysis of photocrosslinking in HJ constructs. (**a**–**c**) Images of denaturing PAGE with samples of HJ constructs tested for photocrosslinking. The electrophoresis was carried out at 57 °C in 1× TBE running buffer. (**a**) Fluorescence image (λ_ex_ = 475 nm, λ_em_ = 537 nm) of SYBR Gold stained gel with the unmodified HJ and T*-labeled HJs. (**b**,**c**) Fluorescence image (λ_ex_ = 632 nm, λ_em_ = 691 nm) of the gel with the SQ-labeled monomers, adjacent dimers and transverse dimers. Non-irradiated samples of the single stranded SQ-labeled DNA (ss SQ-A) and SQ-tetramer tethered to HJ were used as controls. The **SQ-A_2_D_2_** sample was applied in triplicate. (**d**–**f**) Photocrosslinking reaction yield (%) of HJ constructs obtained by quantification of PAGE band intensities.

To induce a photocrosslinking reaction between thymine modifiers, prepared HJ constructs were subjected to UV irradiation at 310 ± 7 nm at room temperature for 60 min. The reaction outcomes were evaluated by denaturing urea polyacrylamide gel electrophoresis (PAGE), which was used to separate ssDNA based on the molecular weight. Under denaturing conditions, an HJ construct lacking a crosslink between opposing strands fully denatured into four single strands, while an HJ construct with a formed crosslink partially denatured into two single strands and a crosslinked double strand exhibiting lower mobility than the single strands. The photocrosslinking yields were estimated from a band intensity ratio of the crosslinked double strand and single strands (Section S2). According to the denaturing PAGE in Figure 3a, no crosslinked product was detected upon irradiation of the unmodified HJ control (Figure 2a).

We started by examining T-T photocrosslinking reactions in the SQ-labeled monomers, where one thymine modifier was covalently tethered to a dichloroindolenine squaraine dye (Figure 2d). Monomer constructs **SQ-A**, **SQ-B**, **SQ-C**, and **SQ-D** produced 5.4%, 10.2%, 3.3%, and 6.9% of photoproducts, respectively (Figure 3b,e). In the control T*-B containing one thymine modifier without the dye, the photocrosslinking yield was 1.6% (Figure 3a,d). The observed low reactivity of the monomers was attributed to the photocrosslinking reaction between the thymine modifier and a nearby pyrimidine (thymine or cytosine) base present in the core of HJ.

Next, we proceeded with examining the photocrosslinking reactions in the squaraine dimers. The yields of photoproduct in the transverse dimers **SQ-AC** and **SQ-BD** were 1.6% and 2.8%, respectively (Figure 3b,e). On the other hand, in the corresponding control T*-AC containing two unlabeled thymine modifiers on the non-complementary strands, the photocrosslinked product was formed with a noticeably higher yield of 16.7% (Figure 3a,d). In the series of adjacent dimers, the **SQ-AD**, **SQ-CD**, **SQ-AB**, and **SQ-BC** constructs exhibited the photocrosslinking yields of 7.0%, 10.1%, 6.3%, and 45.1%, respectively (Figure 3b,e), while the control constructs T*-AD and T*-BC afforded 5.9%, and 1.6%, respectively (Figure 3a,d). The comparable crosslinking yields of photoproducts in **SQ-AD**, **SQ-CD**, and **SQ-AB** to those of monomers (with the exception of **SQ-BC**) suggested that crosslinked photoproducts in dimer constructs might form due to the reaction of thymine modifiers with pyrimidine bases as observed in the monomers.

Taken together, these results showed that the presence of only one unpaired thymine (dye-labeled or unlabeled) in the core of HJ was sufficient for a photocrosslinking reaction with a neighboring pyrimidine base to a small extent (≤10%). Interestingly, the presence of two thymine modifiers did not noticeably improve the outcomes of the photocrosslinking in most of the cases, showing that having two thymine modifiers (dye-labeled or unlabeled) on opposing strands was not sufficient for the photocrosslinking to occur in the HJ in a substantial yield. The results of two distinctive cases of T*-AC and **SQ-BC** with high crosslinking yields suggested that incorporation of thymine modifiers in the HJ core may change the proximity of the thymine modifiers in a favorable or unfavorable way for the T-T [2 + 2] cycloaddition. A very low reaction yield in **SQ-AC,** compared with T*-AC (1.6% vs. 16.7%), suggested that tethering dyes to thymine modifiers in the transverse configuration unfavorably pushed the thymine modifiers further apart. In contrast, tethering the dyes to thymine modifiers in the adjacent configuration in **SQ-BC** dimer resulted in a significantly higher reaction yield compared with the unlabeled T*-BC (45.1% vs. 1.6%) which suggested that thymine modifiers were closer to each other in this construct than in the corresponding T*-BC construct lacking the dyes. Our previous work demonstrated that squaraine dyes covalently tethered to the HJ formed dye aggregates driven by strong attractive Van der Waals interactions that manifested in short intermolecular distances and strong excitonic coupling between the dyes [47]. Such strong dye interactions may further decrease the distance between dye-labeled thymines facilitating [2 + 2] cycloaddition between them [47]. If this is the case, and strong dye interactions promote the proximity of thymine modifiers in the adjacent configuration, we raise a question as to why other squaraine adjacent dimers were not as reactive as **SQ-BC**.

Prompted by our observation that **SQ-BC** produced significantly higher photocrosslinking yield than unlabeled T*-BC and other adjacent dimers with analogous relative placement of thymine modifiers, we examined the **SQ-BC** dimer construct for distinct structural features. We realized that the **SQ-BC** dimer differed from other dimers in having an A-T base pair adjacent to the dye-labeled thymine modifier pair. As such, we hypothesized that the presence of an A-T base pair in the core of the HJ next to the SQ-labeled thymine modifiers played a strong role in the T-T photocrosslinking yield. To test this hypothesis, we replaced the C-G pair next to the thymine modifiers in the **SQ-AD** dimer with an A-T pair. In particular, we modified the sequence of the adjacent dimer **SQ-AD** and designed **SQ-A_2_D_2_** in which the A strand was modified by replacing G with an A base and the D strand was modified by replacing C with a T base near the HJ core (Figure 2e). The dimer **SQ-A_2_D_2_** showed 38.0% photocrosslinking yield compared with the unmodified counterpart whose yield was only 7.0% (Figure 3c,f). We attributed the increase in the reaction yield to the weaker A-T hydrogen bonding strength and thus a lower energy to break the A-T hydrogen bonds which allowed the proximate SQ-labeled thymine modifiers to overlap, adopting a favorable conformation for the [2 + 2] cycloaddition. Another possible explanation was that photocrosslinking reaction could take place laterally between the SQ-labeled thymine modifier and a nearby unlabeled thymine present in the adjacent A-T base pair. 

**Figure 4:**
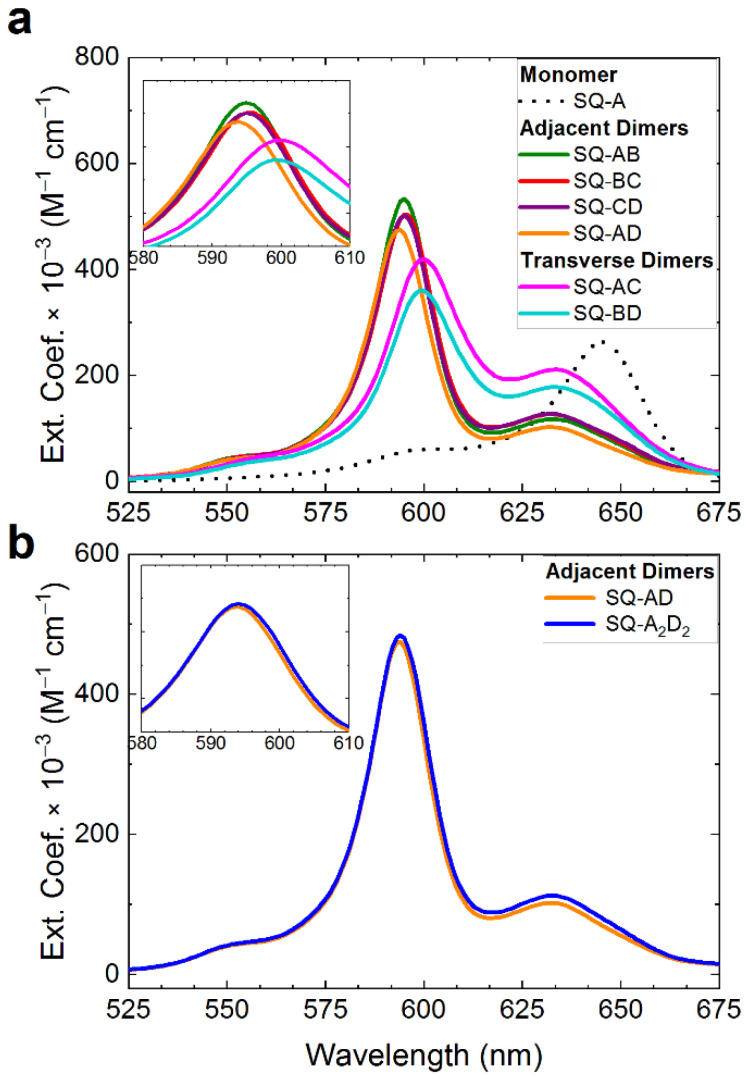
Acquired steady-state absorption spectra recorded in 1× TBE, 15 mM MgCl_2_ at room temperature and converted to extinction of squaraine-DNA HJ constructs. The DNA-dye construct concentration was 1.5 μM. (**a**) Adjacent and transverse dimers (solid lines) and a monomer (dash line). (**b**) Transverse **SQ-AD** and **SQ-A_2_D_2_** dimers.

Next, we proceeded to determine if proximity of squaraine-labeled thymine modifiers in the adjacent and transverse configurations estimated by photocrosslinking experiments correlated with the excitonic coupling strength in the adjacent and transverse dimers. The signatures of excitonic coupling between the dyes could be observed in the spectral changes upon dimer formation with respect to a monomer [26,55]. The excitonic coupling in squaraine dimers studied here was evident in a blue shift of low- and high-energy absorption bands, as well as in the intensification of the high energy band relative to the low energy band (Figure 4 and Figure S4). Based on the blue shift and intensity of high energy absorption band, the adjacent squaraine dimers (**SQ-AB**, **SQ-BC**, **SQ-CD**, and **SQ-AD**) exhibited comparable excitonic coupling strength, which was stronger than that in the transverse dimers (**SQ-AC** and **SQ-BD**). Using modeling of experimental spectral data by the Kühn–Renger–May-based approach [56], the intermolecular center-to-center distances in the representative adjacent **SQ-BC** and transverse **SQ-AC** dimers were determined to be 3.40 Å and 5.45 Å, and the strength of excitonic coupling to be 132 meV and 80 meV, respectively [47]. The high photocrosslinking yields in the adjacent dimers **SQ-BC** and **SQ-A_2_D_2_** versus negligible yields in the transverse dimers indicated that in the adjacent configuration, the thymines were positioned very closely compared with those in the transverse configuration, which likely led to shorter intermolecular dye distance and stronger excitonic coupling. The proximity of thymine-modifiers in the adjacent configuration became detectable by the photocrosslinking method in the presence of the A-T neighboring base pair. A lower dissociation energy of the A-T base pair increased flexibility of the junction and enabled the proximate thymine modifiers to adopt a favorable conformation for the [2 + 2] cycloaddition. The type of neighboring base pair (A-T vs. G-C) did not, however, affect the excitonic coupling strength in the adjacent squaraine dimers as all adjacent dimers exhibited very similar spectral profiles (Figure 4a and Figure S4a). Moreover, the dimers **SQ-AD** and **SQ-A_2_D_2_** that were different only in the nature of the neighboring base pair, exhibited identical spectral profiles (Figure 4b and Figure S4b).

Finally, as the **SQ-BC** dimer demonstrated the highest photocrosslinking reactivity, we examined if the reaction conditions could be further optimized to increase the reaction yield in this construct (Section S4). The examination of the reaction time course showed no increase in yield after 60 min of exposure to UV light. Irradiation at shorter or longer wavelengths than 310 nm resulted in a lower photocrosslinking yield (Figure S5). Irradiation in short time intervals (1600 s) with annealing between the irradiation intervals did not improve the photocrosslinking yield (Figure S6).

### 2.3. Squaraine Tetramer Templated by Doubly Crosslinked HJ

**Figure 5:**
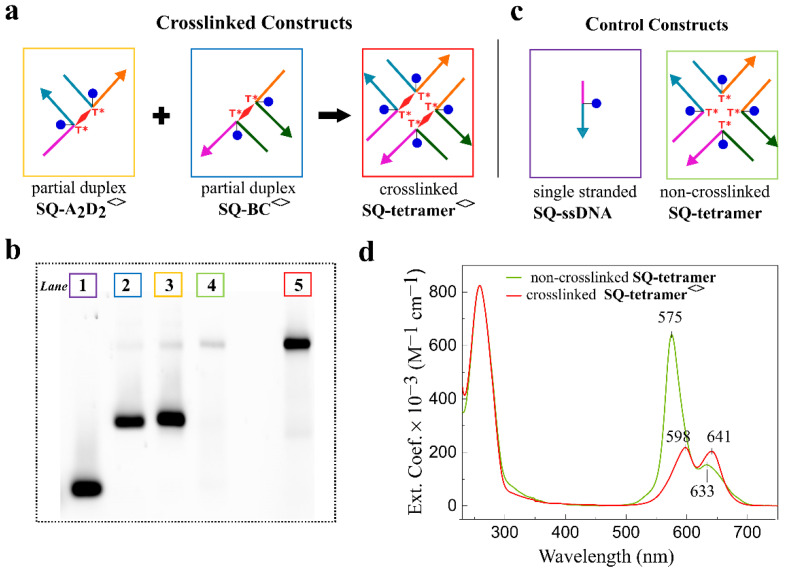
**SQ-tetramer^<>^** templated by a doubly crosslinked HJ. (**a**) Schematic representation of the assembly of **SQ-tetramer^<>^** from purified crosslinked partial duplexes. (**b**) Fluorescence image of non-denaturing PAGE (15%) of squaraine-DNA constructs. Lane numbers are color-coded to the boxes of DNA construct schematics. The samples were applied on the gel at 0.3 µM concentration. Note that band visibility in lane 4 is low due to strongly suppressed fluorescence in the non-crosslinked **SQ-tetramer** aggregate. (**c**) Schematics of the control constructs used to analyze the formation of crosslinked **SQ-tetramer** HJ. (**d**) Acquired steady-state absorption spectra of non-crosslinked **SQ-tetramer** and crosslinked **SQ-tetramer^<>^** in 1× TBE, 15 mM MgCl_2_ at room temperature. The SQ-DNA concentrations were 1.5 μM.

The presence of a covalent bond between opposing strands in the HJ might increase the stability of HJ-templated dye aggregates by presumably mitigating DNA breathing [57]. Covalent bonding between opposing strands in HJ might also decrease heterogeneity by suppressing isomerization of the HJ [58]. To test the utility of crosslinks in creating more stable DNA-templated dye aggregates, we formed crosslinks in **SQ-BC** and **SQ-A_2_D_2_** dimers upon irradiation at 310 ± 7 nm at room temperature for 60 min. The crosslinked constructs in the form of partial duplexes, **SQ-BC^<>^** and **SQ-A_2_D_2_^<>^** (where “<>” stands for a crosslink in the construct), were purified by denaturing PAGE (Figure S7), and were obtained in in the molar amounts of 338 pmole and 435 pmole, respectively, corresponding to 33.8% and 43.4% actual yields, respectively. Thus, the actual yields that accounted for photocrosslinking and recovery from PAGE (Section S6) were similar to the yields determined for **SQ-BC** and **SQ-A_2_D_2_** via electrophoretic analysis. These partial duplexes were combined in 1 to 1 ratio followed by annealing to form an **SQ-tetramer**^<>^ templated by a full HJ containing two crosslinks, i.e., a doubly crosslinked HJ (Figure 5a). The crosslinked partial duplexes **SQ-BC^<>^** and **SQ-A_2_D_2_^<>^**, and the doubly crosslinked **SQ-tetramer^<>^** were characterized by non-denaturing PAGE (Figure 5b), absorption spectroscopy, and thermal denaturation. On the non-denaturing PAGE, the crosslinked **SQ-tetramer^<>^** appeared as a single distinct band exhibiting the same electrophoretic mobility as the band of the control non-crosslinked **SQ-tetramer** (Figure 5b). This result indicated that the presence of the crosslink in each constituent partial duplex **SQ-BC^<>^** and **SQ-A_2_D_2_^<>^** did not inhibit the HJ assembly. 

The absorption properties of the doubly crosslinked **SQ-tetramer^<>^** were compared with those of the non-crosslinked **SQ-tetramer**. In the non-crosslinked **SQ-tetramer**, four squaraines formed a tetramer aggregate that exhibited strong excitonic coupling as reported earlier [47]. The excitonic interaction between the four dyes was evident in the absorption profile of the **SQ-tetramer** characterized by a blue shift of the absorption bands as well as by the increased intensity of the high energy band relative to one of the low energy band in respect to the dimer and monomer (Figure S11). We expected a crosslinked **SQ-tetramer^<>^** to have a similar absorption profile as the non-crosslinked analogue. However, the absorption profile of the crosslinked **SQ-tetramer**^<>^ in the visible region was characterized by broader absorption bands and showed decreased intensity of the high energy band relative to the low energy band compared with that of the non-crosslinked **SQ-tetramer** (Figure 5c). A decrease in dye band intensity relative to the intensity of DNA absorption band at 260 nm was also observed. We attributed these observations to a dye degradation and/or linker cleavage upon UV irradiation which resulted in some strands lacking a dye that led to the decreased presence of not only squaraine tetramers, but trimers, dimers, and monomers in the crosslinked HJ construct. 

The stabilities of the crosslinked partial duplexes **SQ-BC^<>^** and **SQ-A_2_D_2_^<>^,** as well as doubly crosslinked **SQ-tetramer** HJ synthesized by hybridizing crosslinked partial duplexes **SQ-BC^<>^** and **SQ-A_2_D_2_**^<>^ were evaluated by thermal denaturation (Section S7). Crosslinked partial duplexes **SQ-BC^<>^** and **SQ-A_2_D_2_^<>^** exhibited rather complex melting profiles with two extracted melting temperatures of 65.9 °C and 77.1 °C, and 64.7 °C and 76.7 °C, respectively (Table S5, Figure S10). In comparison, the non-crosslinked partial duplexes **SQ-BC** and **SQ-A_2_D_2_** exhibited melting temperatures of 59.3 °C and 58.2 °C, respectively. The increase in melting temperatures of partial duplexes **SQ-BC^<>^** and **SQ-A_2_D_2_^<>^** strongly supported the presence of crosslinks in these constructs. The doubly crosslinked **SQ-tetramer^<>^** showed the melting temperature of 67.4 °C compared with the non-crosslinked tetramer that melted at 66.1 °C. We have previously shown that dye–dye interactions increase melting points of the DNA HJs [47,48]. Smaller than expected increases of the melting temperature of doubly crosslinked **SQ-tetramer^<>^** compared with that of non-crosslinked tetramer could be attributed to weaker dye–dye interaction caused by the partial loss of dyes during irradiation.

## 3. Materials and Methods

### 3.1. Sample Preparation

DNA oligomers functionalized with custom dichloroindolenine squaraine, *N*-hydroxysuccinimide ester of 2-((1-(5-carboxypentyl)-5-chloro-3,3-dimethylindolin-2-ylidene)methyl)-4-((5-chloro-1,3,3-trimethyl-3H-indol-1-ium-2-yl)methylene)-3-oxocyclobut-1-en-1-olate (SETA BioMedical, Urbana-Champaign, IL, U.S.A.) and purified by dual HPLC were purchased from IDT (Integrated DNA Technologies, Inc., Coralville, IA, USA). Non-functionalized DNA oligomers purified by standard desalting were purchased from IDT. All DNA oligomers were rehydrated in ultrapure water (Barnstead Nanopure, Thermo Scientific, Waltham, MA, USA) to obtain 100 μM stock solutions. The concentrations of stock DNA samples were validated spectroscopically on NanoDrop One Microvolume UV-Vis (Thermo Scientific) using theoretical extinction coefficients. DNA HJ constructs were prepared by combining equimolar amounts of partially complementary oligomers in 1× TBE, 15 mM MgCl_2_ buffer solution to obtain a 1.5 μM final DNA concentration. All DNA samples were annealed in Mastercycler Nexus PCR cycler (Eppendorf) according to the following protocol: 4 min at 94 °C, followed by cooling to room temperature (22 °C). 

### 3.2. Photocrosslinking Reaction

The samples were placed in the Horiba PTI QuantaMaster 400 spectrofluorometer (Horiba Scientific, Piscataway, NJ, USA) equipped with a xenon short arc 18 V 450 W lamp (Ushio, Tokyo, Japan). DNA samples in 1× TBE, 15 mM MgCl_2_ in a 500 μL Eppendorf vial were irradiated at 310 ± 7 nm at room temperature for 60 min. 

### 3.3. Analytical Denaturing Gel Electrophoresis

PAGE (15%) gel electrophoresis was performed to evaluate the outcomes of photocrosslinking reactions in DNA HJ constructs. Denaturing electrophoresis gels, 1.5 mm thick, were prepared in 1× TBE, 20% urea and pre-run at 57 °C, 600 V for 30 min. DNA samples were diluted 10-fold in loading buffer (formamide: 100× TBE, 9:1 *v*/*v*) and denatured at 95 °C for 4 min prior to application on a gel. The DNA samples were loaded on the PAGE gel, and the electrophoresis was carried out for 25 min at 350 V applied voltage and at 57 °C constant temperature in running buffer 1× TBE. The electrophoresed gels were washed with ultrapure water (3 × 200 mL). Unlabeled DNA samples were stained with SYBR gold for 30 min. The gels were placed onto a phosphor plate, and imaged in FluorChem Q imager (Alpha Innotech, San Leandro, CA, USA) using the Cy5-channel (excitation 632 nm: emission 691 nm) for squaraine-labeled DNA samples or the Cy2-channel (excitation 475 nm: emission 506 nm) for unlabeled SYBR gold-stained DNA samples. The raw gels (i.e., without contrast and brightness adjustments) were analyzed in ImageJ 1.53e [59] (Section S2).

### 3.4. Synthesis of SQ-Tetramer Templated by Doubly Crosslinked HJ

The dimer **SQ-BC** (20 µM, 50 µL) in 1× TBE, 15 mM MgCl_2_, was irradiated for 60 min at room temperature at 310 ± 7 nm wavelength in a Horiba PTI QuantaMaster 400 spectrofluorometer (Horiba Scientific) equipped with a 450 W xenon lamp. DNA samples were combined with equal volume loading buffer (formamide: 100× TBE, 9:1 *v*/*v*) and heated at 95 °C for 4 min to denature the DNA prior to loading on the gel in six wells (Figure S7). The electrophoresis was run at 350 V applied voltage at 56 °C constant temperature for 25 min in running buffer 1× TBE. The bands of crosslinked and non-crosslinked products were excised separately. The excised bands were transferred into Freeze ‘N Squeeze spin columns (Bio-Rad, Hercules, CA, USA), carefully crushed, and incubated at −20 °C for 15 min. Samples were spun down at 13,000 rpm for 3 min to collect the filtrate. The gel debris was soaked in 250 µL of 1× TBE, 15 mM MgCl_2_ overnight at 4 °C, then spun to collect the filtrate. Combined filtrates were transferred to the Amicon centrifugal tubes for concentration and buffer exchange. Crosslinked samples were placed in 10 kD MWCO Amicon tube, whereas non-crosslinked samples were placed in 3 kD MWCO Amicon tube. All crosslinked and non-crosslinked samples were subjected to concentration followed by buffer exchange into fresh 1× TBE, 15mM MgCl_2_. The samples were recovered by inverting the inner chamber into a fresh collecting tube and spinning at 2000 rpm for 2 min. The dimer **SQ-A_2_D_2_** was subjected to the analogous procedure of irradiation and purification. The concentrations of isolated crosslinked partial duplexes **SQ-BC^<>^** and **SQ-A_2_D_2_^<>^** were determined on NanoDrop using the theoretical extinction coefficient (ε = 468,094 M^−1^cm^−1^) to afford 5.63 μM; 60 μL (338 pmoles), and 7.9 μM; 55 μL (435 pmoles) of the partial duplex **SQ-BC^<>^** and **SQ-A_2_D_2_^<>^**, respectively. The actual yields including photocrosslinking and photoproduct recovery were 33.8% and 43.4% for the partial duplexes **SQ-BC^<>^** and **SQ-A_2_D_2_^<>^**, respectively.

*Absorption*. UV-Vis spectra of the DNA-dye aggregates at 1.5 μM concentration in 1× TBE, 15 mM MgCl_2_ buffer were recorded in duplicate at room temperature in a quartz cuvette with 1 cm path length (Starna) in a 230–800 nm of wavelength range using a dual-beamed Cary 5000 UV–Vis–NIR spectrophotometer (Agilent Technologies, Santa Clara, CA, USA). 

## 4. Conclusions

In this work, we investigated the structural characteristics of squaraine-HJ dimers that lead to strong excitonic coupling between dyes. By performing photocrosslinking of thymine-modifiers tethering the dyes, we demonstrated for the first time that covalent crosslinks between opposing thymines could form in the four-arm DNA HJ. We further found that three structural characteristics were necessary to achieve high photocrosslinking yield in squaraine dimers templated by the HJ. These components, which work in synergy, were: (1) placement of dye-labeled thymine modifiers on the partially complemented strands in the immobile HJ, (2) the presence of an A-T base pair next to the pair of thymine modifiers, and (3) chemical structure of the dyes capable of exhibiting strong dye–dye interactions to increase proximity of thymine-modifiers. The results from the photocrosslinking experiments strongly support the notion that intermolecular distances and excitonic coupling strength in squaraine dimers, which are favorable for the photocrosslinking reaction, stem from the proximity of the thymine modifiers tethering the dyes to the core of the HJ. The practical implication of our work is that covalent bonds between thymine modifiers, i.e., crosslinks, can be utilized in creating more stable, and, in principle, less heterogeneous HJ-templated dye aggregates, which we demonstrated with the dichloroindolenine squaraine tetramers covalently templated by the doubly crosslinked HJ. In this regard, future work will be focused on: (1) decreasing the detrimental effect of irradiation by optimizing the parameters of UV irradiation, using scavengers of reactive oxygen species, and utilizing multiple or shorter linkers for the attachment of the dyes, and (2) applying our photocrosslinking-based aggregate formation approach to larger nanostructures in order to achieve higher order stable aggregates. 

## Data Availability

The data presented in this study are contained within the article and Supplementary Material.

## Supplementary Materials

The following supporting information can be downloaded at: https://www.mdpi.com/article/10.3390/molecules27134006/s1, DNA sequences, electrophoretic analysis, photocrosslinking reaction optimization, non-denaturing PAGE, thermal denaturation, and spectral measurements. Ref [59] is cited in the Supplementary Materials.

## Sample Availability

Samples of the compounds are not available from the authors.
