# Supplementary material for: Photocrosslinking Probes Proximity of Thymine Modifiers Tethering Excitonically Coupled Dye Aggregates to DNA Holliday Junction
Source: Molecules. 2022 Jun 22;27(13):4006. doi: 10.3390/molecules27134006 (PMC9268628; doi:10.3390/molecules27134006)
Supplement: Supplementary file 1 [file molecules-27-04006-s001.zip › molecules-1775034-original image.pdf]

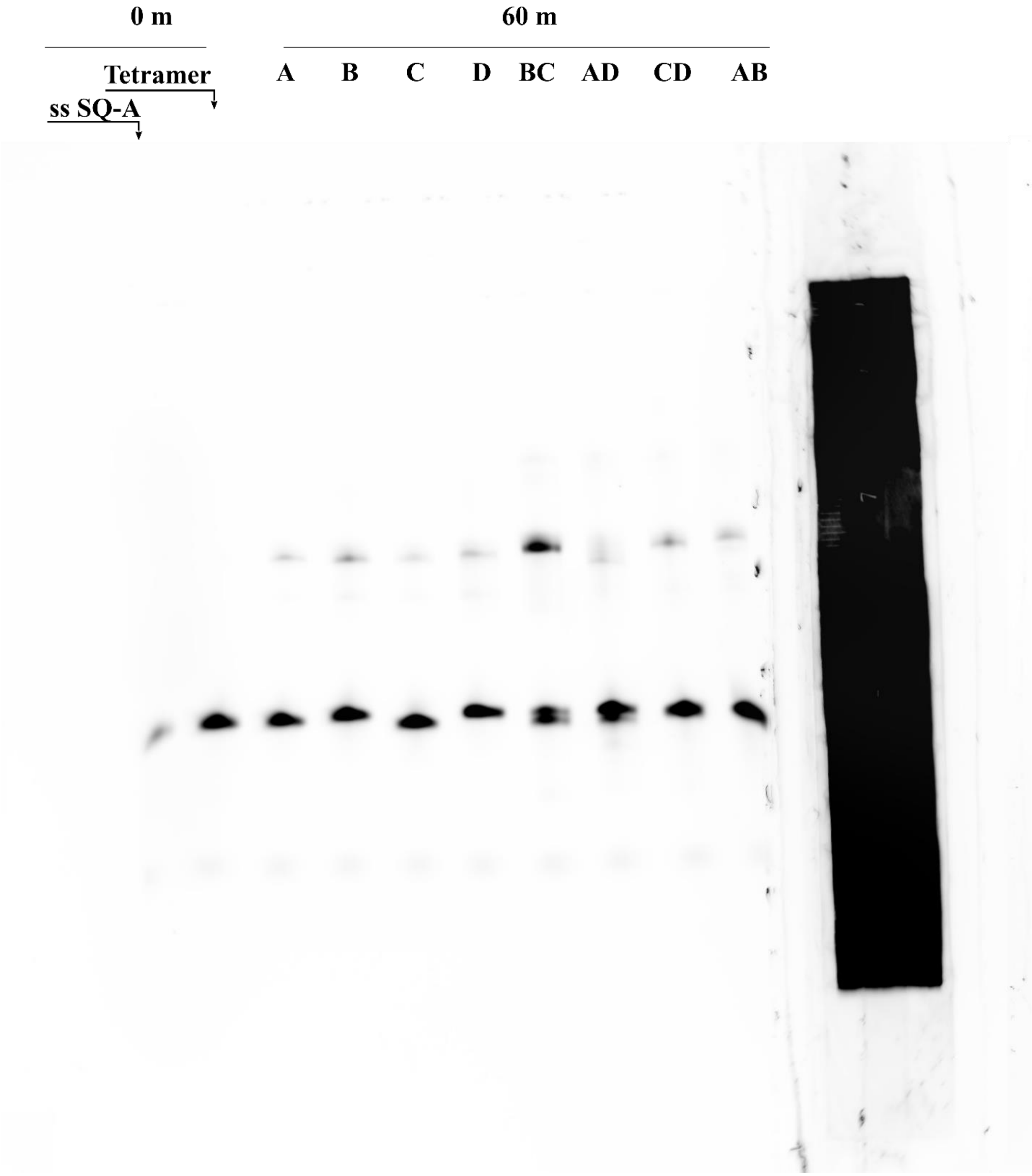

Figure 3b (left)/ Figure S2 (left). Fluorescent image (ex. 632 nm, em. 691 nm) of non-stained 15% denaturing PAGE, 1.5 mm-thick. Lane 1: single-stranded SQ-A DNA, 0 min irradiation; lane 2: SQ-tetramer template by HJ, 0 min irradiation; lanes 3-6: squaraine monomers SQ-A, SQ-B, SQ-C and SQ-D, 60 min irradiation; lanes 7-10: squaraine adjacent dimers. SQ-BC, SQ-AD, SQ-CD, and SQ-AB, 60 min irradiation.

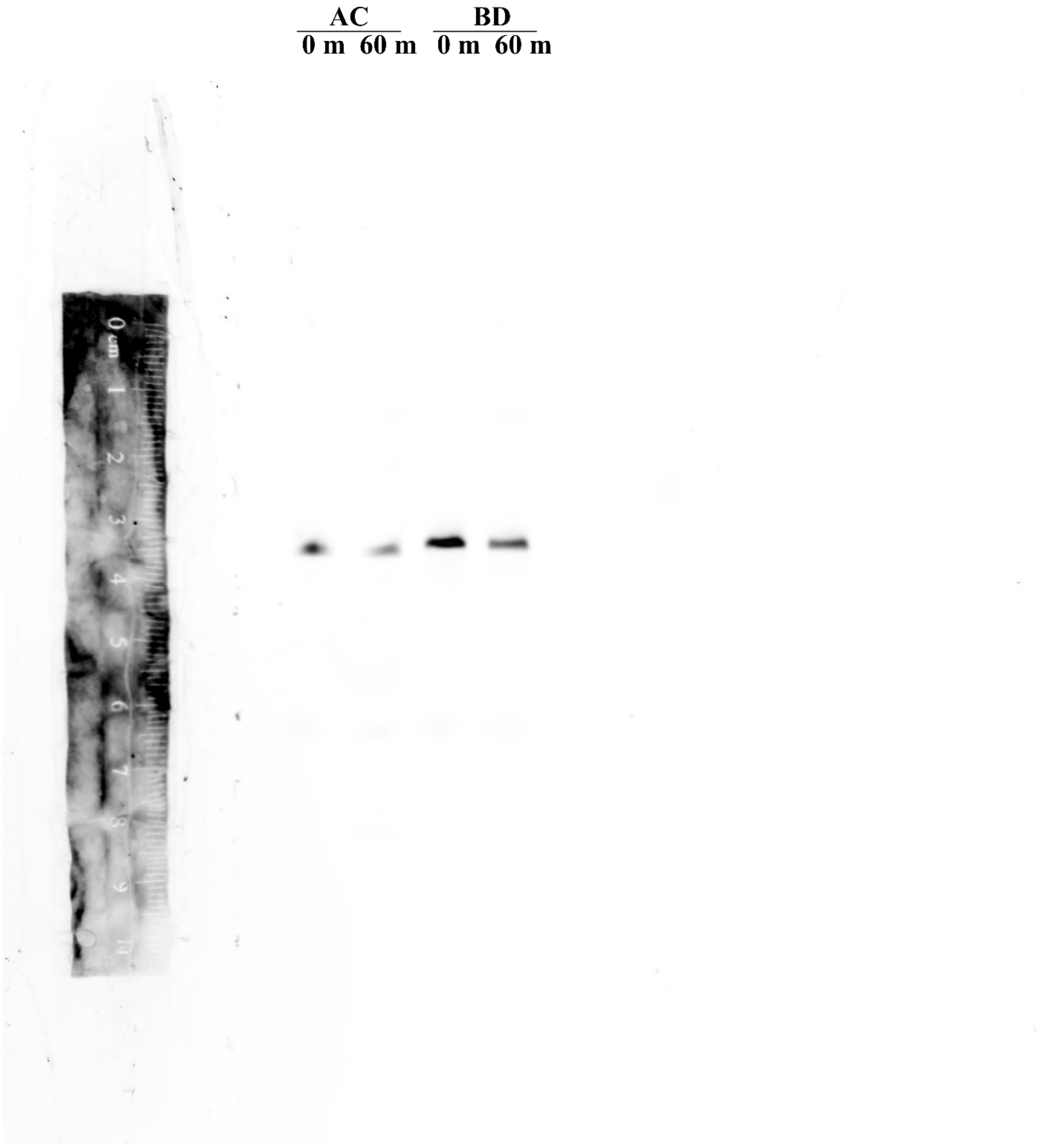

Figure 3b (right)/ Figure S2 (right). Fluorescent image (ex. 632 nm, em. 691 nm) of non-stained 15% denaturing PAGE, 1.5 mm-thick. Lane 1: squaraine transverse dimer SQ-AC, 0 min irradiation; lane 2: squaraine transverse dimer SQ-AC, 60 min irradiation; lane 3: squaraine transverse dimer SQ-BD, 0 min irradiation; lane 4: squaraine transverse dimer SQ-BD, 60 min irradiation.

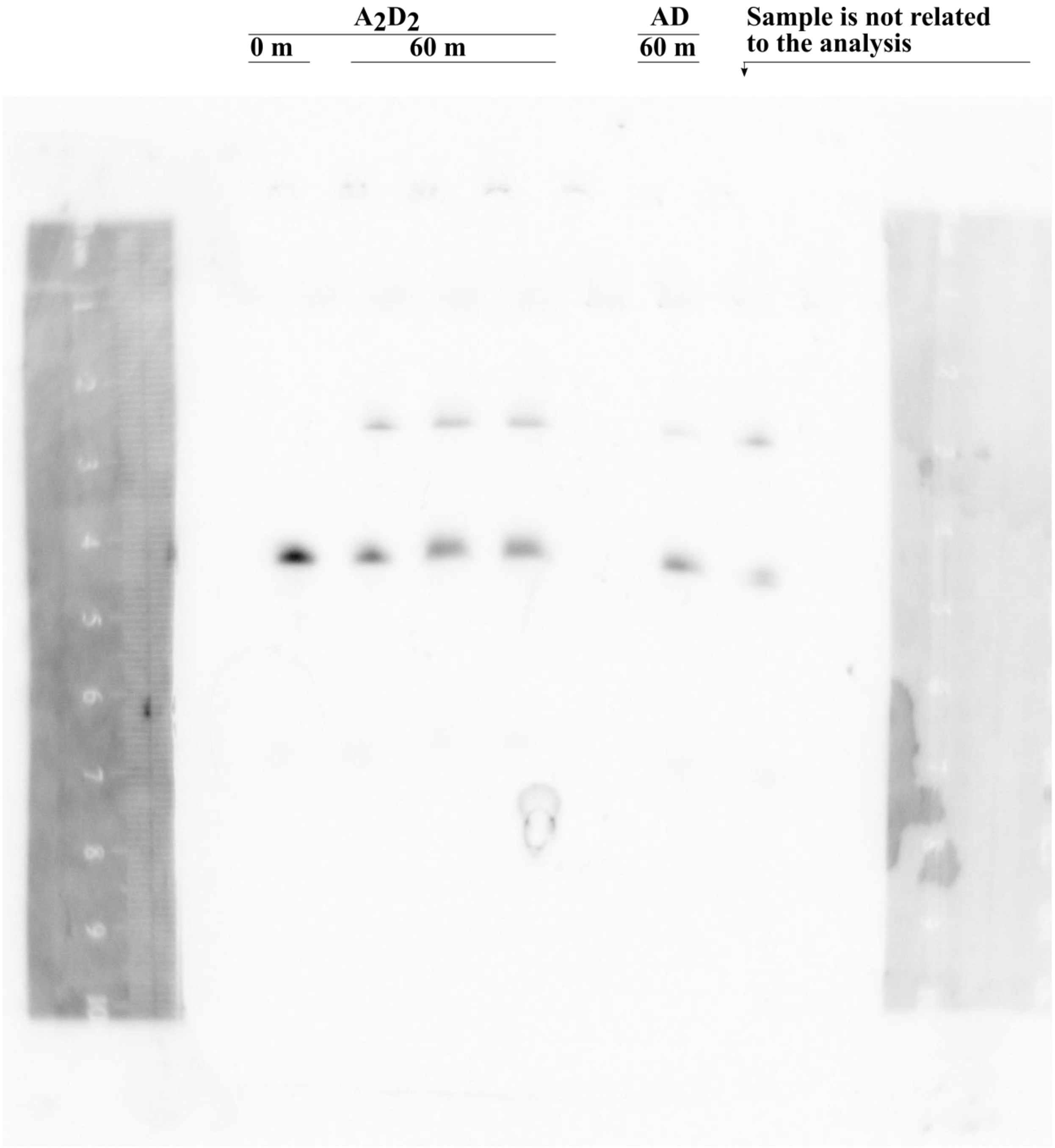

Figure 3c/ Figure S2. Fluorescent image (ex. 632 nm, em. 691 nm) of non-stained 15% denaturing PAGE, 1.5 mm thick. Lane 1: squaraine adjacent dimer SQ-A_2D_2, 0 min irradiation. Lanes 2-4: squaraine adjacent dimer SQ-A_2D_2, 60 min irradiation applied in triplicate; lane 5: empty; lane 6: squaraine adjacent dimer SQ-AD, 60 min irradiation; lane 7: sample unrelated to this project.

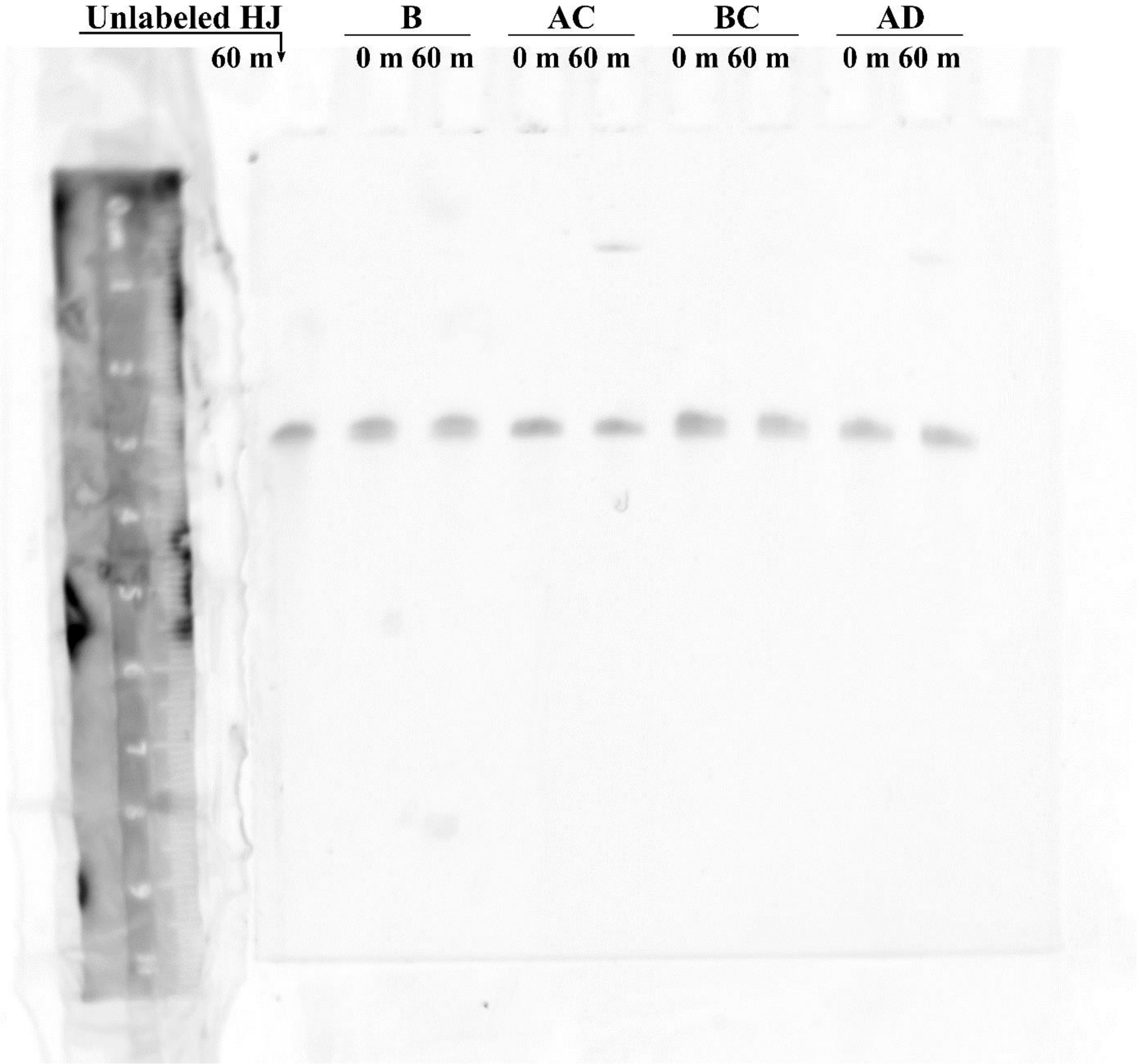

Figure 3a/ S3. Fluorescent image (ex. 475 nm, em. 537 nm) of 15% denaturing PAGE, 1.5 mm thick, stained with SYBR Gold, of HJ constructs containing unlabeled thymine modifier(s). Lane 1: unmodified HJ, 60 min irradiation; lane 2: **T*-B**, 0 min irradiation; lane 3: **T*-B**, 60 min irradiation; lane 4: **T*-AC**, 0 min irradiation; lane 5: **T*-AC**, 60 min irradiation; lane 6: **T*-BC**, 0 min irradiation; lane 7: **T*-BC**, 60 min irradiation; lane 8: **T*-AD**, 0 min irradiation; lane 9: **T*-AD**, 60 min irradiation.

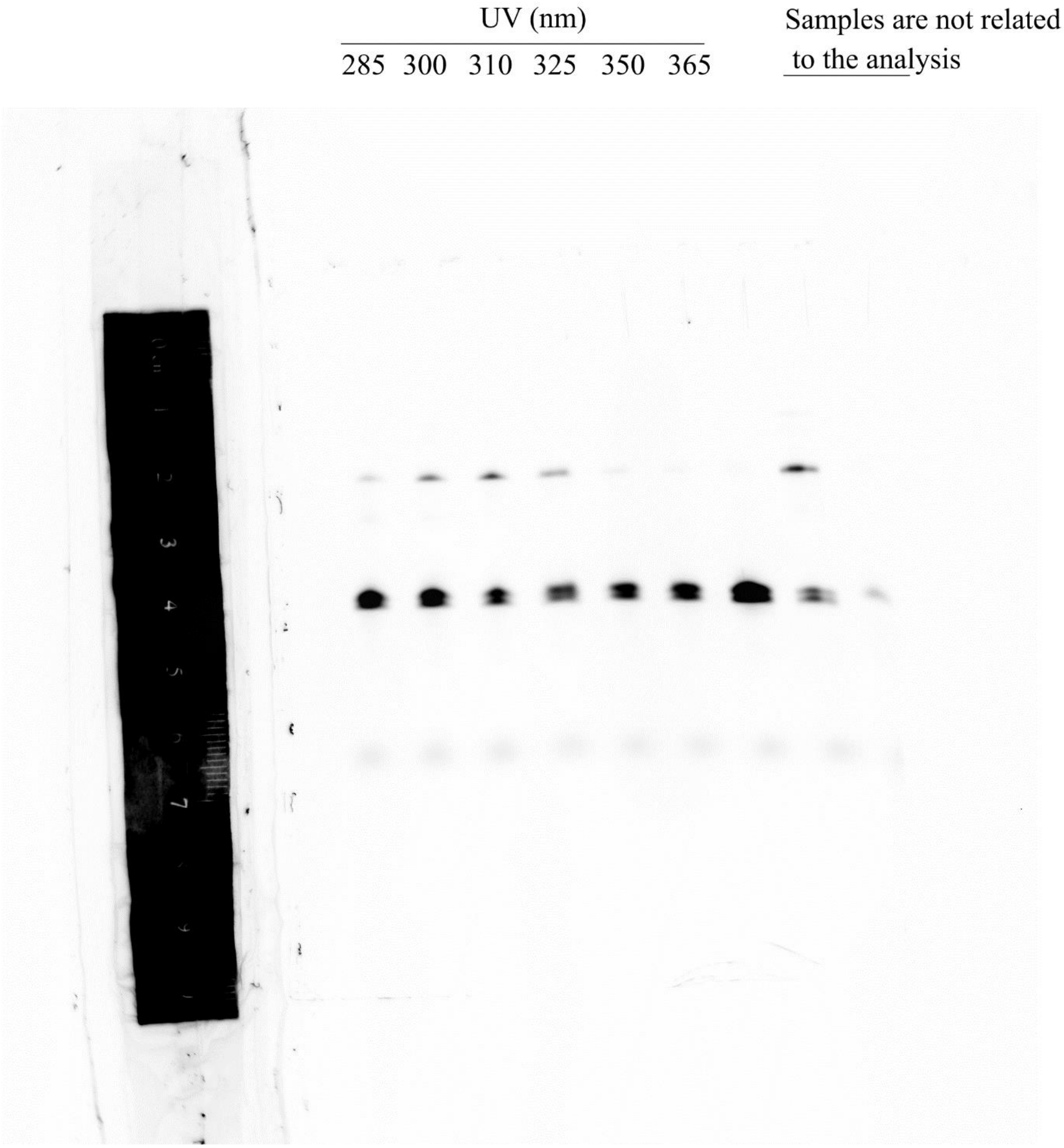

Figure S5a. Fluorescence image (ex. 632 nm, em. 691 nm) of non-stained denaturing PAGE, 1.5 mm thick, of photocrosslinking reactions in **SQ-BC** construct at different wavelengths. Lane 1: 285 nm; lane 2: 300 nm; lane 3: 310 nm; lane 4: 325 nm, lane 5: 350 nm; lane 6: 365 nm.

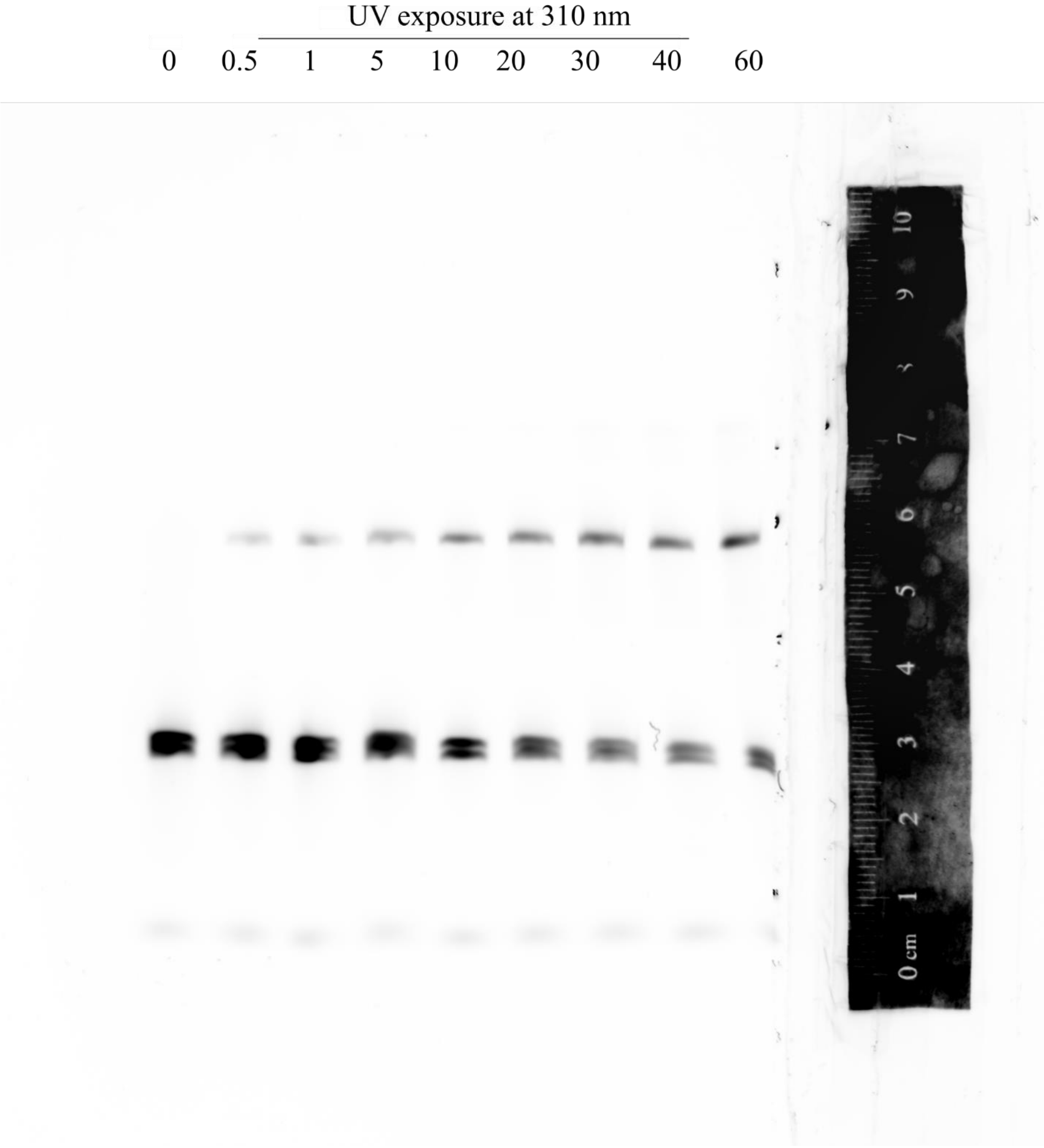

Figure S5b. Fluorescence image (ex. 632 nm, em. 691 nm) of non-stained denaturing PAGE, 1.5 mm thick, of photocrosslinking reaction progress in **SQ-BC** construct at different time points. Lane 1: 0 min; lane 2: 30 sec' lane 3: 1 min; lane 4: 5 min; lane 5: 10 min; lane 6: 30 min; lane 7: 40 min; l lane 8: 60 min.

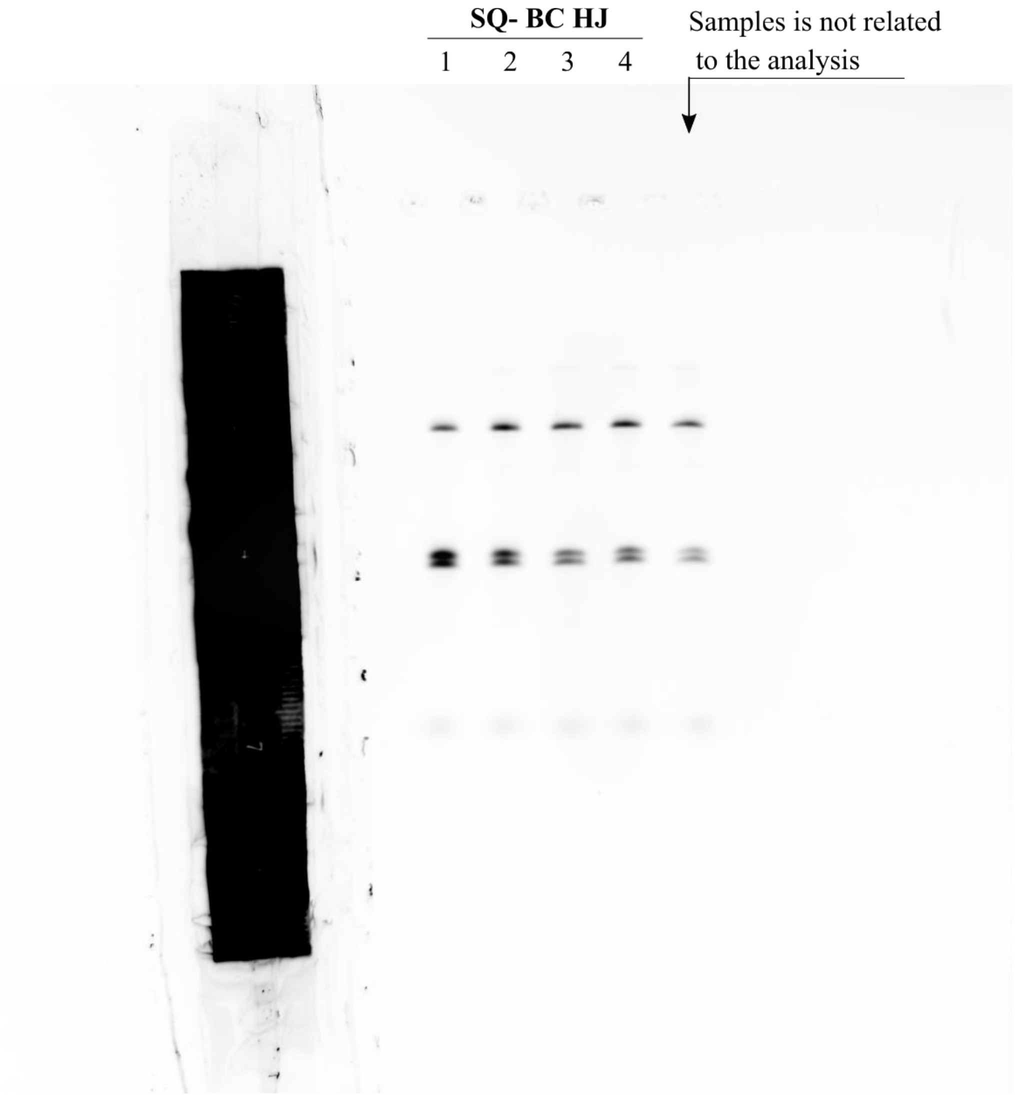

Figure S6a. Fluorescence image (ex. 632 nm, em. 691 nm) of denaturing PAGE, 1.5 mm thick of photocrosslinking progress in **SQ-BC** dimer via a repeated annealing-irradiation (310 nm) cycle. Lane 1: 1600 s; lane 2: 3200 s; lane 3: 4800 s; lane 4: 6400 sec.

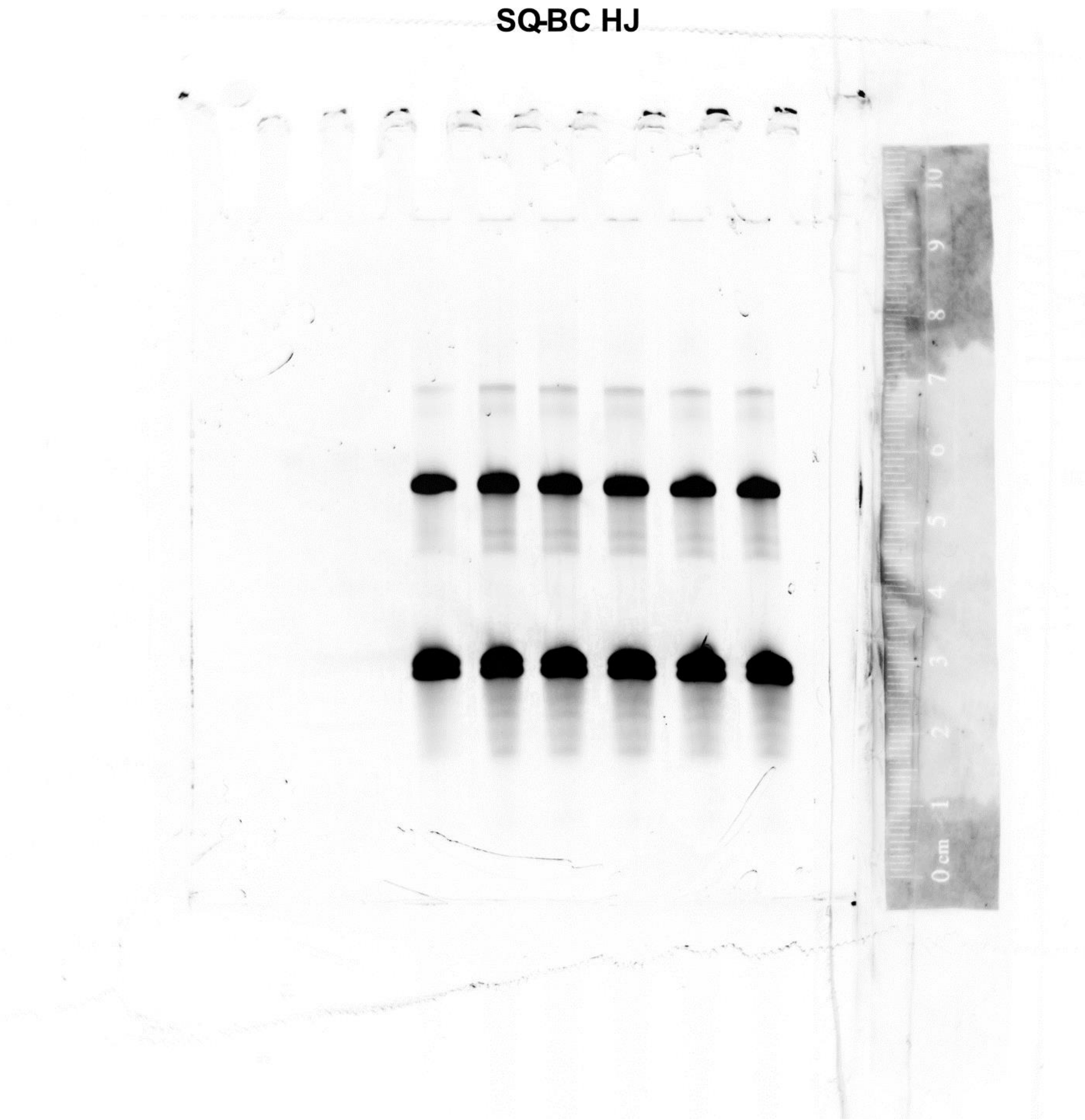

Figure S7 (left). Fluorescence image (ex. 632 nm, em. 691 nm) of denaturing PAGE, 1.5 mm thick of purification of crosslinked **SQ-BC**<> construct. Lanes 1-6: SQ-BC after irradiation at 60 min.

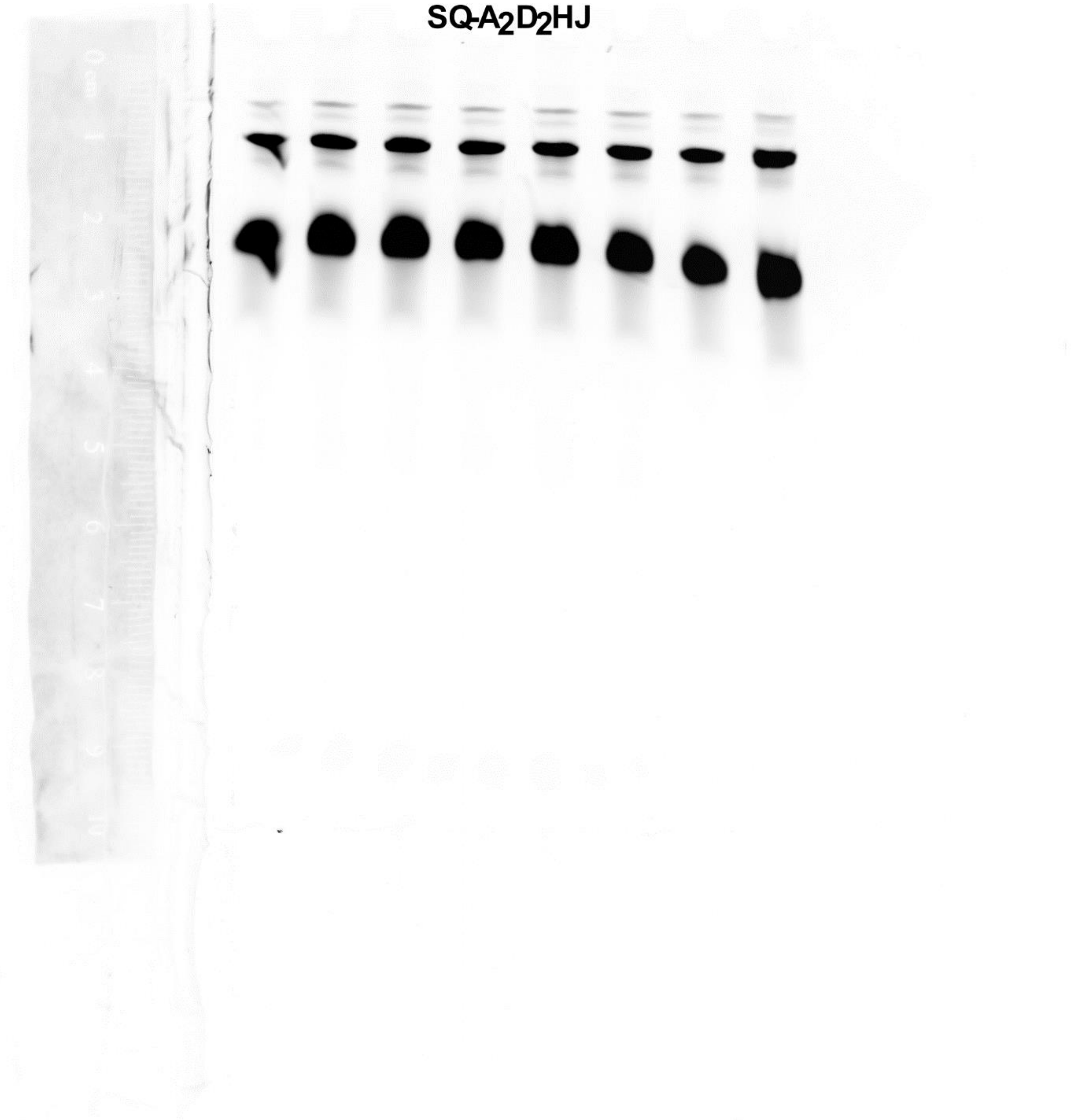

Figure S7 (right). Fluorescence image (ex. 632 nm, em. 691 nm) of denaturing PAGE, 1.5 mm thick of purification of crosslinked **SQ-A$_2$D$_2$**$^{<>}$ construct. Lanes 1-6: SQ-A$_2$D$_2$ after irradiation at 60 min.

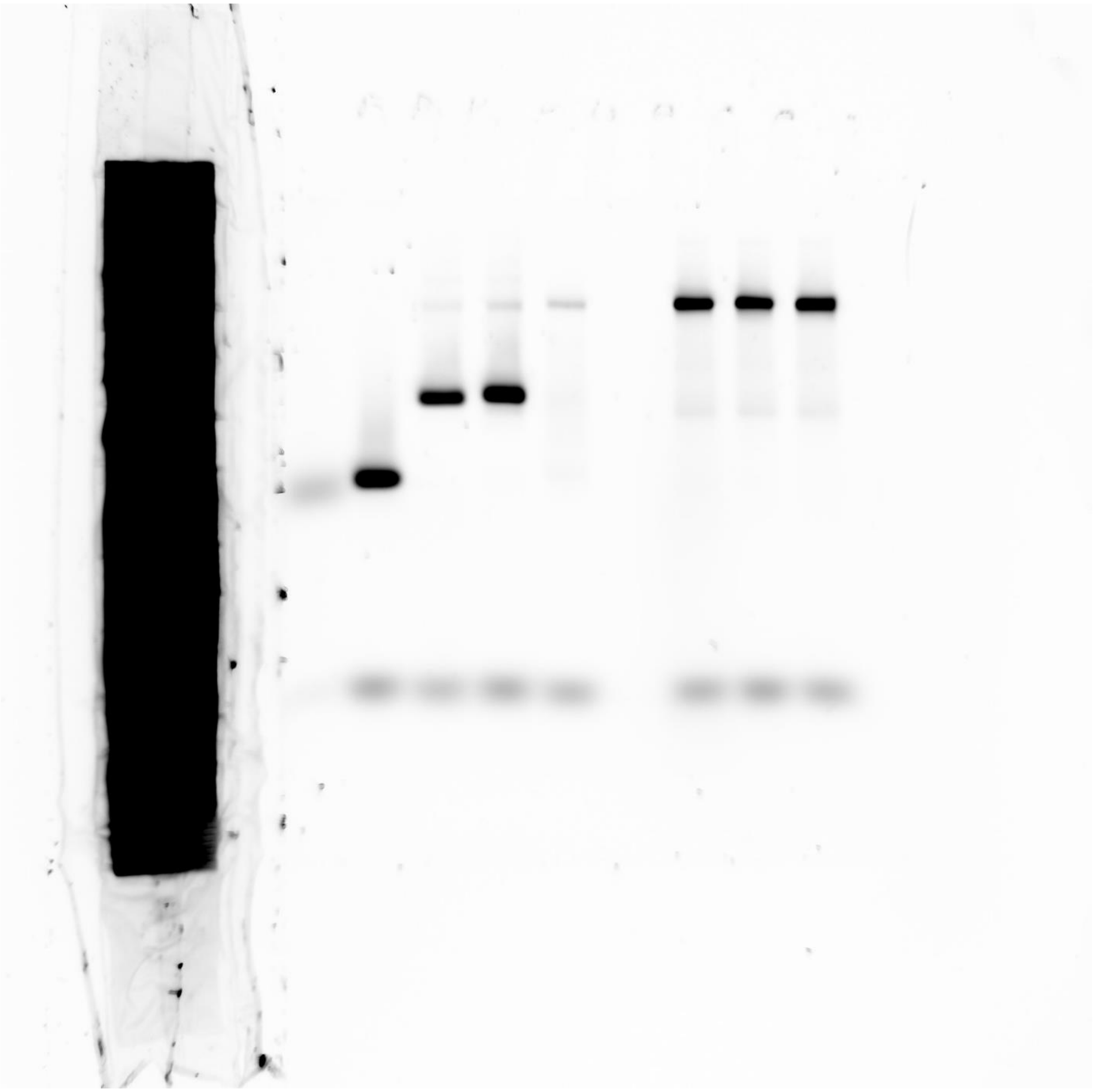

Figure 5b/S8. Fluorescence image (ex. 632 nm, em. 691 nm) of non-stained non-denaturing PAGE (15%) gel electrophoresis of squaraine-DNA constructs. Lane 1: molecular ladder; lane 2: SQ-A ssDNA; lane 3: crosslinked partial duplex SQ-BC<>; lane 4: crosslinked partial duplex SQ-A_2D_2<>; lane 5: non-crosslinked SQ-tetramer; lane 6: empty; lanes 7-9: doubly crosslinked SQ-tetramer<> applied in triplicate.
