# Supplementary material for: Photocrosslinking Probes Proximity of Thymine Modifiers Tethering Excitonically Coupled Dye Aggregates to DNA Holliday Junction
Source: Molecules. 2022 Jun 22;27(13):4006. doi: 10.3390/molecules27134006 (PMC9268628; doi:10.3390/molecules27134006)
Supplement: Supplementary file 1 [file molecules-27-04006-s001.zip › molecules-1775034-SM.pdf]

Supporting Information

Photocrosslinking Probes Proximity of Thymine Modifiers Tethering Excitonically Coupled Dye Aggregates to DNA Holliday Junction

[1]Shibani Basu, [1]Keitel Cervantes-Salguero, [1,2]Bernard Yurke, [1,2]William B. Knowlton, [1,3]Jeunghoon Lee, [1]Olga A. Mass**

[1]Micron School of Materials Science & Engineering, [2]Department of Electrical & Computer Engineering, [3]Department of Chemistry and Biochemistry, Boise State University, Boise, Idaho 83725, United States

Corresponding authors:
*jeunghoonlee@boisestate.edu
*olgamass@boisestate.edu

Table of Contents

SI1	*DNA sequences and nanostructures*	S2
SI2	*Electrophoretic analysis of crosslinked DNA constructs*	S4
SI3	*Absorption spectra of non-crosslinked squaraine dimers*	S8
SI4	*Optimization of photocrosslinking reaction conditions*	S9
SI5	Synthesis and *purification of crosslinked SQ-labeled partial duplexes*	S11
SI6	*Analytical non-denaturing gel electrophoresis of SQ-tetramer templated by double crosslinked HJ.*	S11
SI7	*Thermal denaturation*	S12
SI8	*Spectral properties of SQ-labeled DNA constructs*	S15
SI9	*References*	S17

Supporting Information 1: *DNA sequences and nanostructures*

The DNA oligomer sequences used to assemble immobile unmodified HJ, as well as modified HJ nanostructures are given in **Table S1**. Unmodified DNA oligomers and T*-modified DNA oligomers purified by standard desalting were purchased from IDT (Integrated DNA Technologies, Inc.). A custom dichloroindolenine squaraine dye was obtained from SETA BioMedicals (Urbana-Champaign, IL). Labeling of oligomers with dichloroindolenine squaraine via NHS-ester chemistry and subsequent purification by dual HPLC was performed at IDT (Integrated DNA Technologies, Inc.). The schematics of the assembled HJs are shown in **Figure S1**.

Table S1: DNA oligomer sequences.

Strand Name	[a]Sequence (5' to 3')	[b]Length (nt)
A	ATATAATCGCTCGCATATTATGACTG	26
B	CAGTCATAATATGTGGAATGTGAGTG	26
C	CACTCACATTCCACTCAACACCACAA	26
D	TTGTGGTGTTGAGCGAGCGATTATAT	26
T*-A	ATATAATCGCTCG[T]CATATTATGACTG	27
T*-B	CAGTCATAATATG[T]TGGAATGTGAGTG	27
T*-C	CACTCACATTCCA[T]CTCAACACCACAA	27
T*-D	TTGTGGTGTTGAG[T]CGAGCGATTATAT	27
SQ-A	ATATAATCGCTCG[T*-SQ] CATATTATGACTG	27
SQ-B	CAGTCATAATATG[T*-SQ] TGGAATGTGAGTG	27
SQ-C	CACTCACATTCCA[T*-SQ] CTCAACACCACAA	27
SQ-D	TTGTGGTGTTGAG[T*-SQ] CGAGCGATTATAT	27
SQ-A_2	TATAATCGCTCA[T*-SQ] CATATTATGACTG	27
SQ-D_2	TTGTGGTGTTGAG[T*-SQ] TGAGCGATTATAT	27

[a]The single stranded DNAs constituting HJs are labeled A, B, C and D and the complementary regions of ssDNA are color-coded. [b]Oligomer lengths in terms of the number of nucleotides.

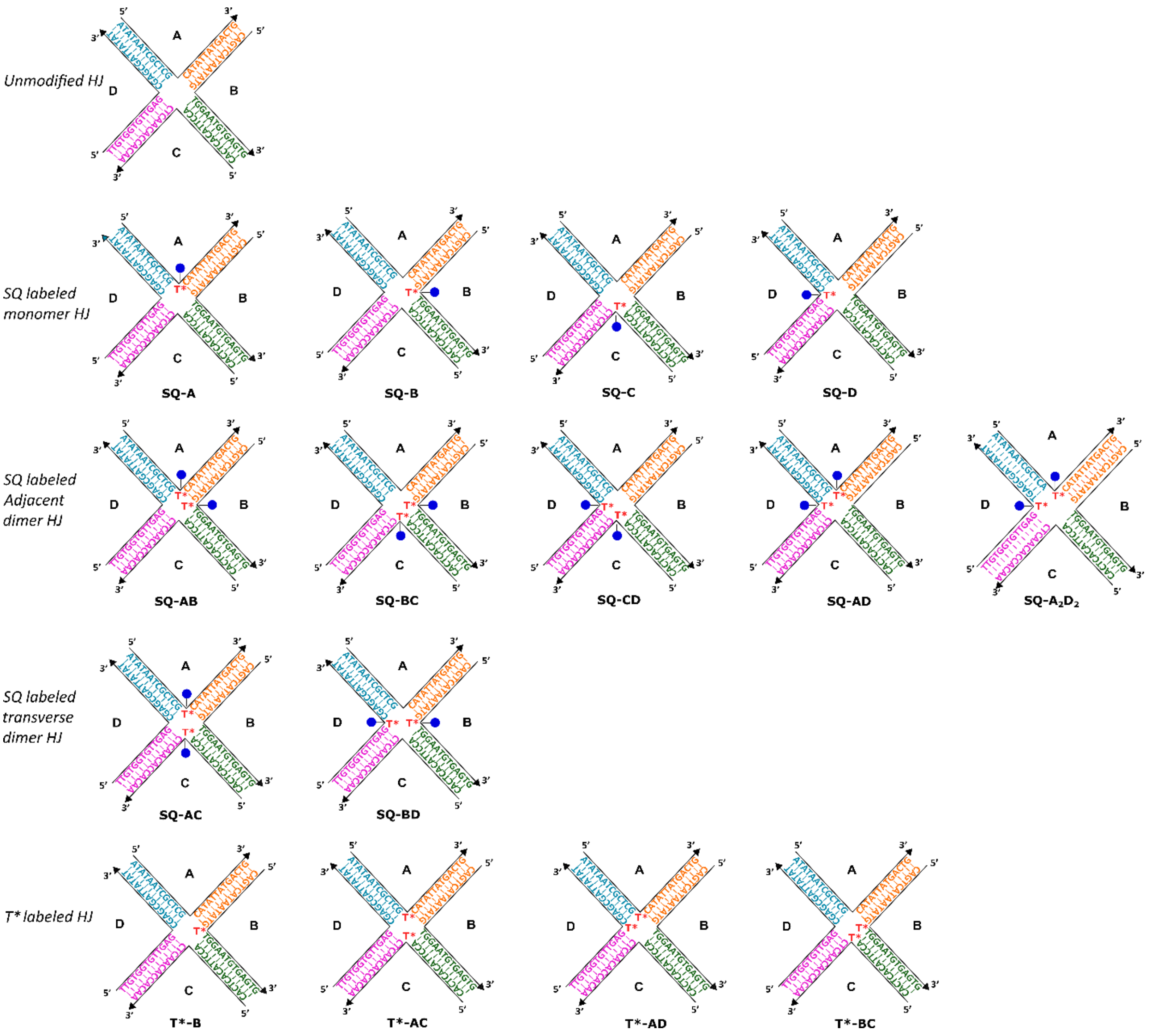

Figure S1. DNA nanostructure designs of immobile HJs. The single stranded DNAs constituting HJs are labeled A, B, C and D, and the complementary regions of ssDNA are color-coded. In unmodified HJ, no thymine modifier is incorporated in the center of HJ. In the modified HJs, incorporated unlabeled and dye-labeled thymine modifiers are depicted respectively as T* and T*- "blue circle".

Supporting Information 2: *Electrophoretic analysis of crosslinked DNA constructs*

Raw gels (i. e. without contrast and brightness adjustments) were analyzed in ImageJ 1.53e.[1]The background was subtracted (50 pixels) (**Figures S2** and **S3**). Photocrosslinking yield was calculated as follows:

$$Photocrosslinking\ Yield\ [\%] = \frac{A_C}{A_C +\ A_{NC}} \times 100$$

where A_C is the area under the peak corresponding to the band of crosslinked DNA; A_{NC} is the area under the peak corresponding to band of the non-crosslinked DNA.

The photocrosslinking yields are summarized in **Tables S2** and **S3.**

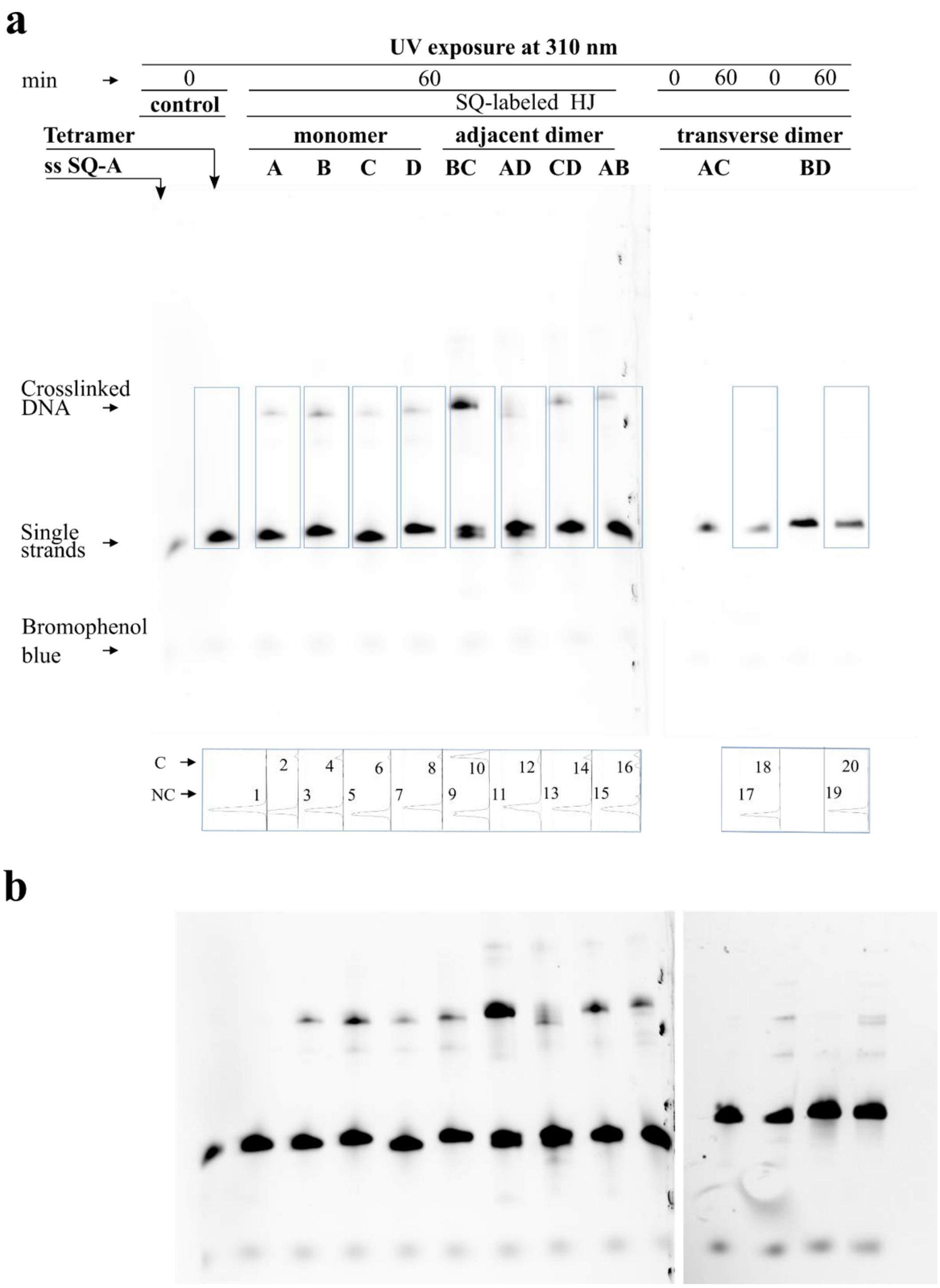

Figure S2. Fluorescent images (ex. 632 nm: em. 691 nm) of 15% denaturing PAGE, 1.5 mm-thick, of squaraine monomers, squaraine adjacent dimers and squaraine transverse dimers. The electrophoresis was performed at 57 °C in 1× TBE running buffer. A single stranded SQ-A DNA and SQ-tetramer HJ were applied as controls. (a) Quantification of the raw gel in ImageJ showing lane selections and corresponding peak areas. (b) Brightness and contrast adjustment of the raw gel to increase visibility of the bands.

Table S2: Yield of crosslinked SQ-DNA HJ analyzed by ImageJ.

Sample (T*-labeled HJ)	Peak #	Peak Area		$A_C/(A_C + A_{NC})$, %
		^aA_C	$^bA_{NC}$	
**A**	1		10177	5.4
	2	582		
**B**	3		10177	10.2
	4	1162		
**C**	5		10590	3.3
	6	351		
**D**	7		9575	6.9
	8	716		
**BC**	9		10366	45.1
	10	8526		
**AD**	11		12344	7.0
	12	937		
**CD**	13		11016	10.1
	14	1247		
**AB**	15		13647	6.3
	16	926		
**AC**	17		12114	1.6
	18	200		
**BD**	19		10427	0.8
	20	86		

^{a}**A_C** - peak area corresponding to the band of the crosslinked DNA; ^{b}**A_{NC}** – peak area corresponding to the band of the non-crosslinked DNA.

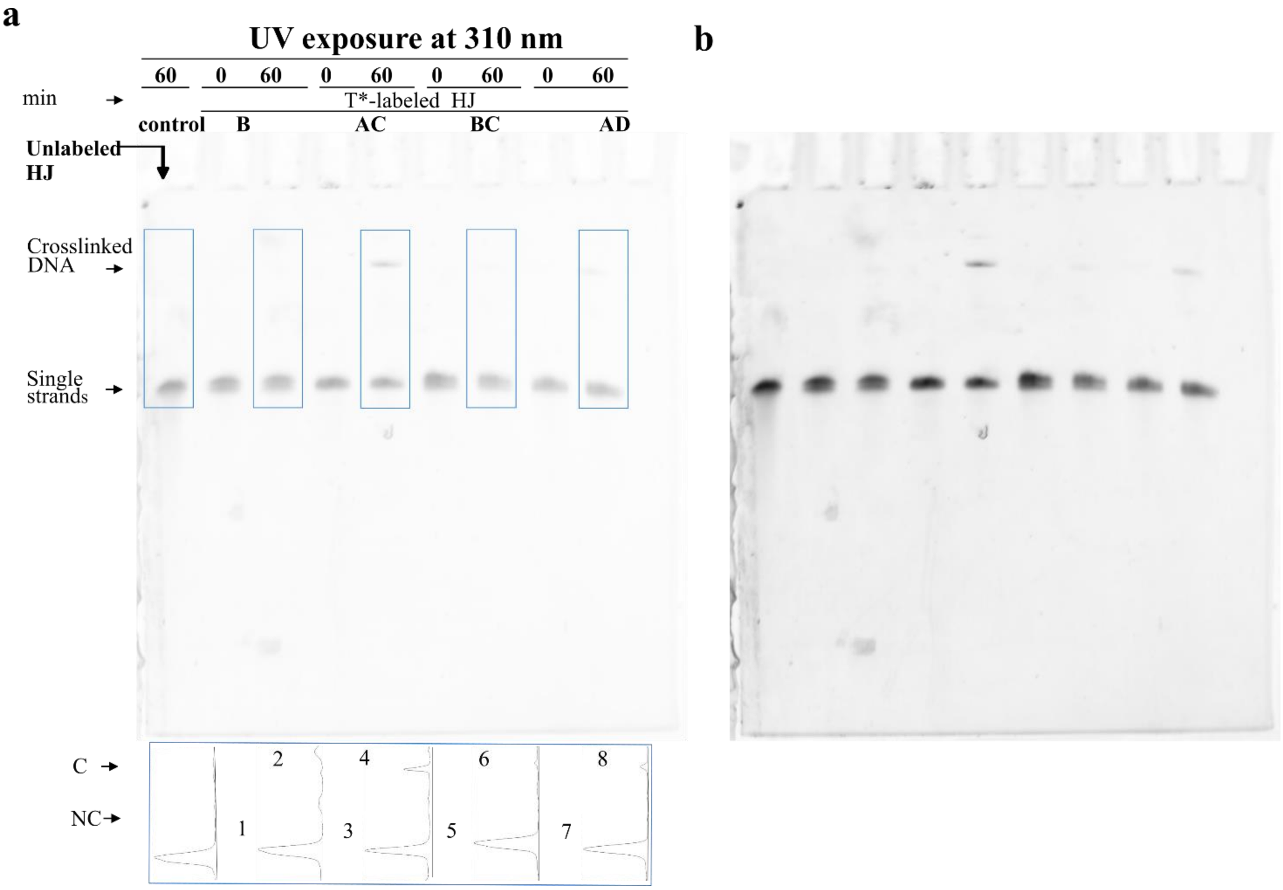

Figure S3. Fluorescent images (ex. 475 nm: em. 537 nm) of 15% denaturing PAGE, 1.5 mm thick, of **T*-B**, **T*-AC**, **T*-BC** and **T*-AD** HJ constructs containing unlabeled thymine modifier(s). The electrophoresis was performed at 57 °C in 1× TBE running buffer. A sample of unmodified HJ was applied as a control. (a) Quantification of the raw gel in ImageJ showing lane selections and corresponding peak areas. (b) Brightness and contrast adjustment of the raw gel to increase visibility of the bands.

Table S3. Yield of crosslinked T*-labeled HJ analyzed by ImageJ.

**Sample (T*-labeled HJ)**	**Peak #**	**Peak Area** ^{a}**A$_C$**	^{b}**A$_{NC}$**	**A$_C$/(A$_C$ + A$_{NC}$), %**
**B**	1		22550	1.6
	2	387		
**AC**	3		14511	16.7
	4	2930		
**BC**	5		18172	1.6
	6	313		
**AD**	7		16934	5.9
	8	1013		

[a]**A$_C$** - peak area corresponding to the band of the crosslinked DNA; [b]**A$_{NC}$** – peak area corresponding to the band of the non-crosslinked DNA.

Supporting Information 3: *Absorption spectra of non-crosslinked squaraine dimers*

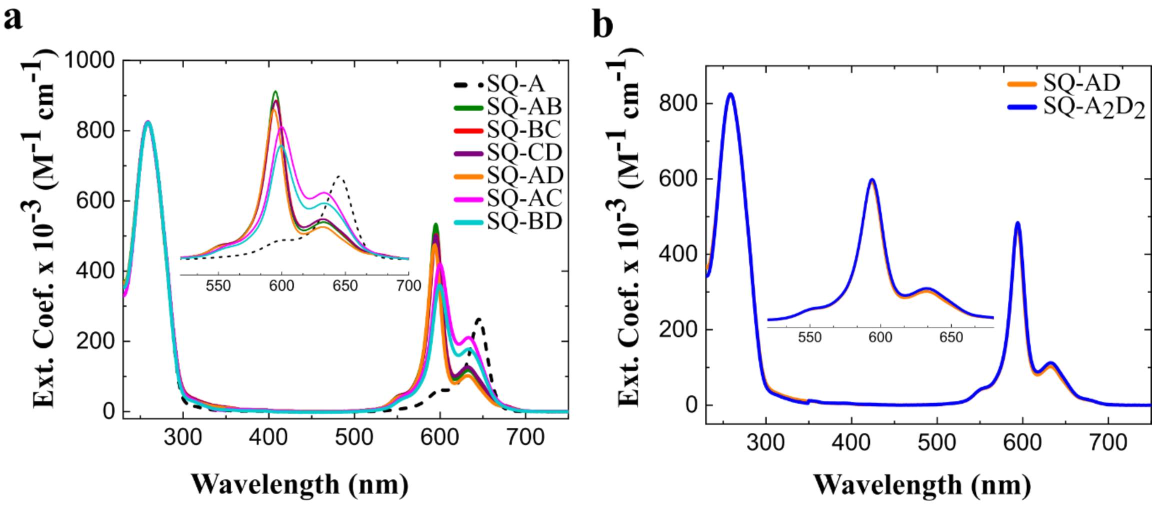

Figure S4. Acquired steady-state absorption spectra converted to extinction of squaraine-DNA HJ constructs in 1× TBE, 15 mM $MgCl_2$ at room temperature. The DNA-dye construct concentration was 1.5 µM. (a) Adjacent and transverse dimers (solid lines) and a monomer (dash line). (b) Transverse **SQ-AD** and **SQ-A_2D_2** dimers.

Table S4. Absorption of non-crosslinked SQ dimers in 1× TBE, 15 mM $MgCl_2$.

SQ dimer HJ	Squaraine absorption peak maximum, nm
**SQ-AB**	595; 632
**SQ-BC**	595; 633
**SQ-CD**	595; 632
**SQ-AD**	594; 633
**SQ-AC**	600; 633
**SQ-BD**	599; 634

Supporting Information 4: *Optimization of photocrosslinking reaction conditions*

In the course of establishing our photocrosslinking method, we attempted to optimize the photocrosslinking yield by examining such reaction conditions as irradiation wavelength and time of UV-exposure. To determine optimal irradiation wavelength, **SQ-BC** dimer was irradiated at 285 nm, 300 nm, 310 nm, 325 nm, 350 nm, and 365 nm. The photocrosslinking yields were determined via electrophoretic analysis as described in Section S2. Irradiation at 310 nm afforded the largest crosslinking yield (**Figure S5**). Next, we evaluated the time course of **SQ-BC** dimer photocrosslinking at 310 nm by taking reaction aliquots (2 μL) at 0.5 min, 1 min, 5 min, 10 min, 20 min, 30 min, 40 min and 60 min. After 30 min of irradiation, the progress of the reaction was observed to be modest.

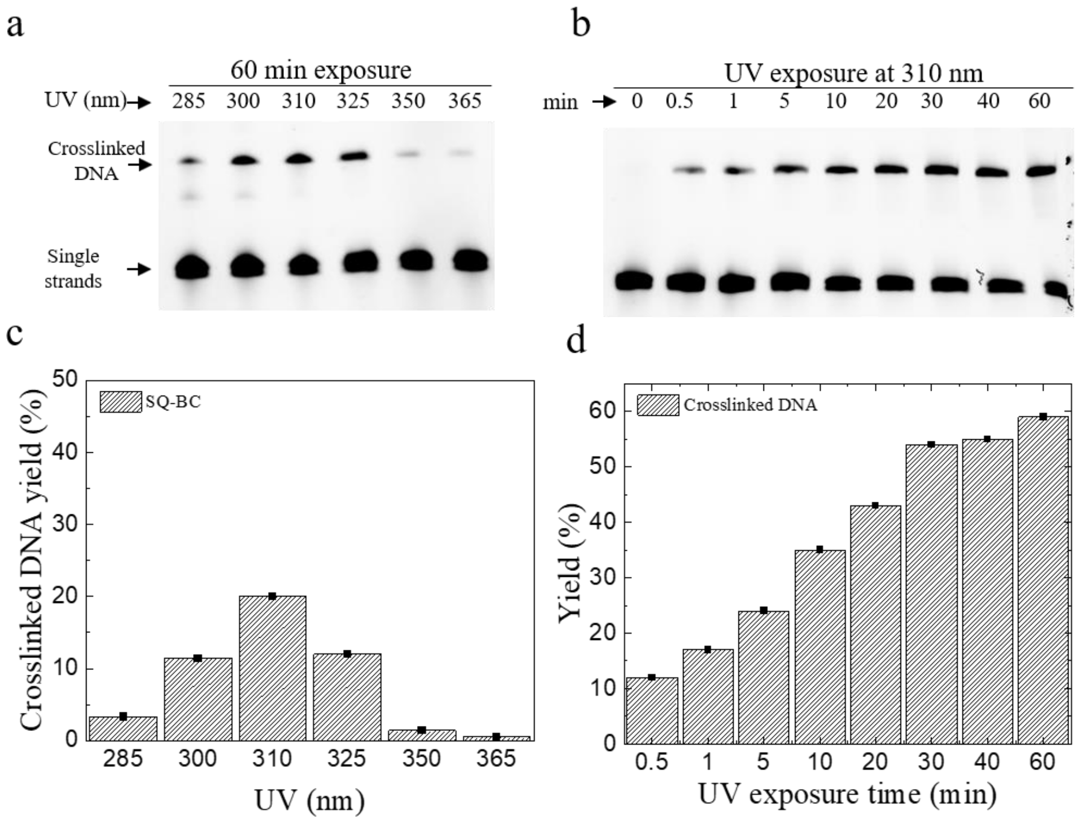

Figure S5. (a, b) Fluorescence images (ex. 632 nm: em. 691 nm) of denaturing PAGE gels of photocrosslinking reactions in **SQ-BC** construct. The electrophoresis was performed at 57 °C in 1× TBE running buffer. (**a**) Photoirradiation at various wavelengths for 60 min. (**b**) Photoirradiation time course for **SQ-BC** dimer. (**c**, **d**) Photocrosslinking reaction yield (%) of **SQ-BC** at different time points obtained by quantification of PAGE band intensities.

In attempt to increase the photocrosslinking yield, we examined photocrosslinking of **SQ-BC** dimer via a repeated annealing-irradiation cycle. In particular, after the initial annealing of **SQ-BC** (4 min at 94 °C, followed by cooling to room temperature), it was irradiated for 1600 s at 310 nm. Then the sequence of annealing - irradiation was repeated three more times for a total of four annealing-irradiation cycles. A reaction aliquot (2 μL) was obtained at the end of each cycle, and photocrosslinking yield at the end of each cycle was determined by the electrophoretic analysis of denaturing PAGE as described in Section S2 (**Figure S6**). The photocrosslinking yield after the fourth cycle of irradiation was calculated to be 45%.

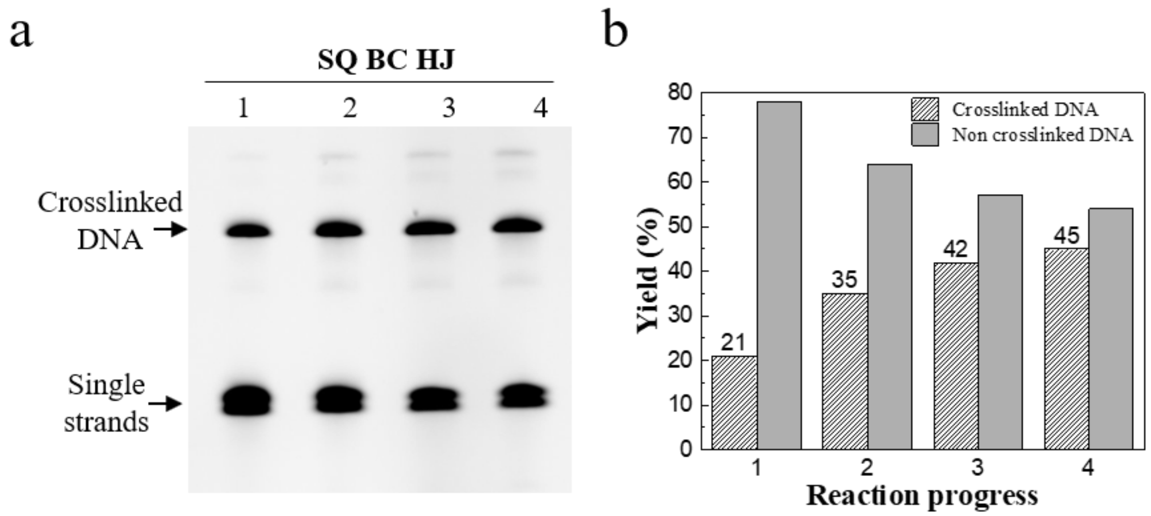

Figure S6. Analysis of photocrosslinking progress in **SQ-BC** dimer via a repeated annealing-irradiation (310 nm) cycle. (a) Fluorescence image (ex. 632 nm: em. 691 nm) of 15% denaturing PAGE gel developed at 57 °C in 1× TBE, 15 mM $MgCl_2$ running buffer. (b) Photocrosslinking yield (%) in **SQ-BC** dimer at the end of each irradiation cycle calculated from electrophoretic analysis. Lane 1: reaction aliquot after 1,600 s of UV exposure, 21% yield; lane 2: reaction aliquot after 3,200 s UV exposure, 35%; lane 3: reaction aliquot after 4,800 s of UV exposure, 42% yield; lane 4: reaction aliquot after 6400 s UV exposure, 45% yield.

Supporting Information 5: *Synthesis and purification of crosslinked SQ-labeled DNA constructs*

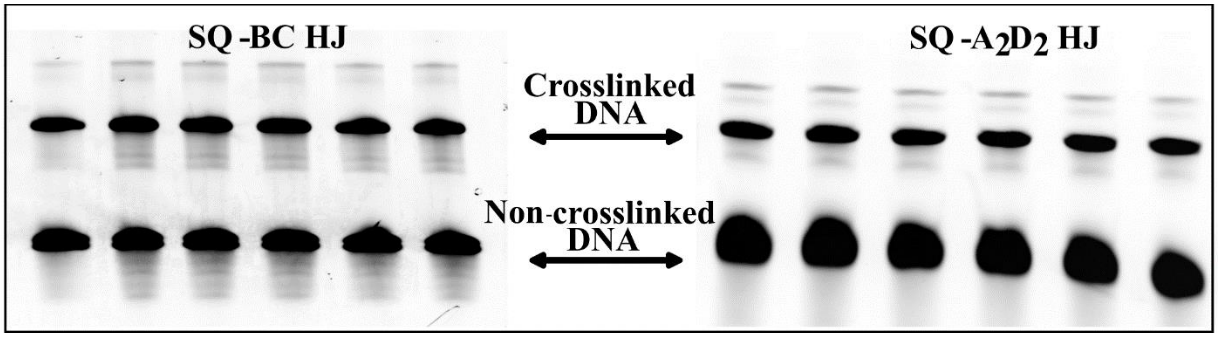

Figure S7. Purification of crosslinked **SQ-BC** and **SQ-A_2D_2** dimers on a denaturing 15% PAGE, 1.5 mm thick, developed in 1× TBE running buffer at 57 °C. Each sample was applied in six gel wells. The developed gel was imaged in the Cy5-channel (ex. 632 nm: em. 691 nm) to determine locations of the bands containing crosslinked and non-crosslinked DNA, after which the bands were excised and subjected to further purification.

Supporting Information 6: *Analytical non-denaturing gel-electrophoresis of SQ-tetramer templated by double crosslinked HJ*

A non-denaturing electrophoresis was performed to analyze the formation of SQ-tetramer templated by doubly crosslinked HJ via hybridization of constituent crosslinked partial duplexes. A non-denaturing 15% PAGE gel, 1.5 mm-thick, was casted in 1× TBE, 15 mM $MgCl_2$ buffer. DNA samples were combined with loading buffer [20% v/v Ficoll (Sigma-Aldrich) and 20% v/v bromophenol blue (Sigma-Aldrich)] to the final concentration 0.3 µM. The electrophoresis was carried out at 17 °C, 150V applied voltage in 1× TBE, 15mM $MgCl_2$ running buffer. The gel was imaged in FluorChem Q imager (Alpha Innotech, San Leandro, CA) using the ex. 632 nm /em. 691 nm channel.

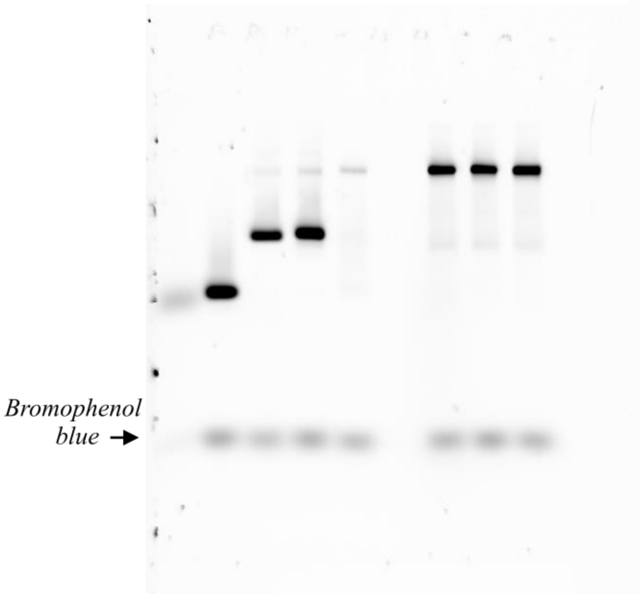

Figure S8. Fluorescence image (ex. 632 nm, em. 691 nm) of non-stained non-denaturing PAGE (15%) gel electrophoresis of squaraine-DNA constructs. Lane 1: molecular ladder; lane 2: SQ-A ssDNA; lane 3: crosslinked partial duplex SQ-BC$^{<>}$; lane 4: crosslinked partial duplex SQ-$A_2D_2^{<>}$; lane 5: non-crosslinked SQ-tetramer; lane 6: empty; lanes 7-9: doubly crosslinked SQ-tetramer$^{<>}$ applied in triplicate.

Supporting Information 7: *Thermal denaturation*

Melting profiles of unmodified HJ and SQ-labeled HJ constructs were recorded on a Cary5000 spectrophotometer equipped with a thermal probe (Agilent Technologies Cary Temperature Controller G9808). Prior the measurement, samples were degassed for 2 min at room temperature. Samples were equilibrated at 25 °C for 5 min before initiating a temperature ramp of 1 °C/min in the 25 °C to 95 °C temperature range. Absorption was monitored at 260 nm. Thermal denaturation temperatures (T_m) of the unmodified HJ and squaraine-DNA constructs were determined by fitting a Gaussian to the first derivative of the absorption profile in OriginPro 2021 **(Figures S9 and S10**). The melting temperatures of unmodified HJ and SQ-labeled DNA are summarized in **Table S5.**

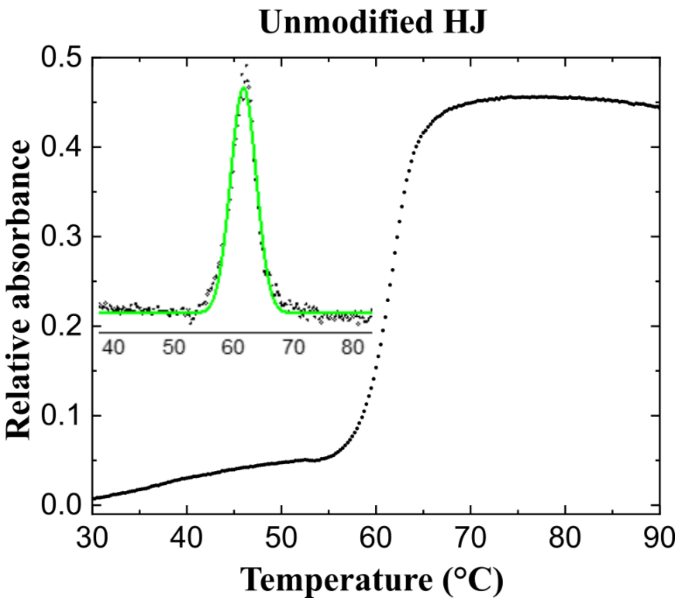

Figure S9. Melting profile of unmodified non-crosslinked HJ in 1× TBE, 15 mM $MgCl_2$. The sample concentration was 1.5 μM. The thermal denaturation was monitored via absorption at 260 nm. The insert shows the first derivative as a function of temperature and a fitted Gaussian curve.

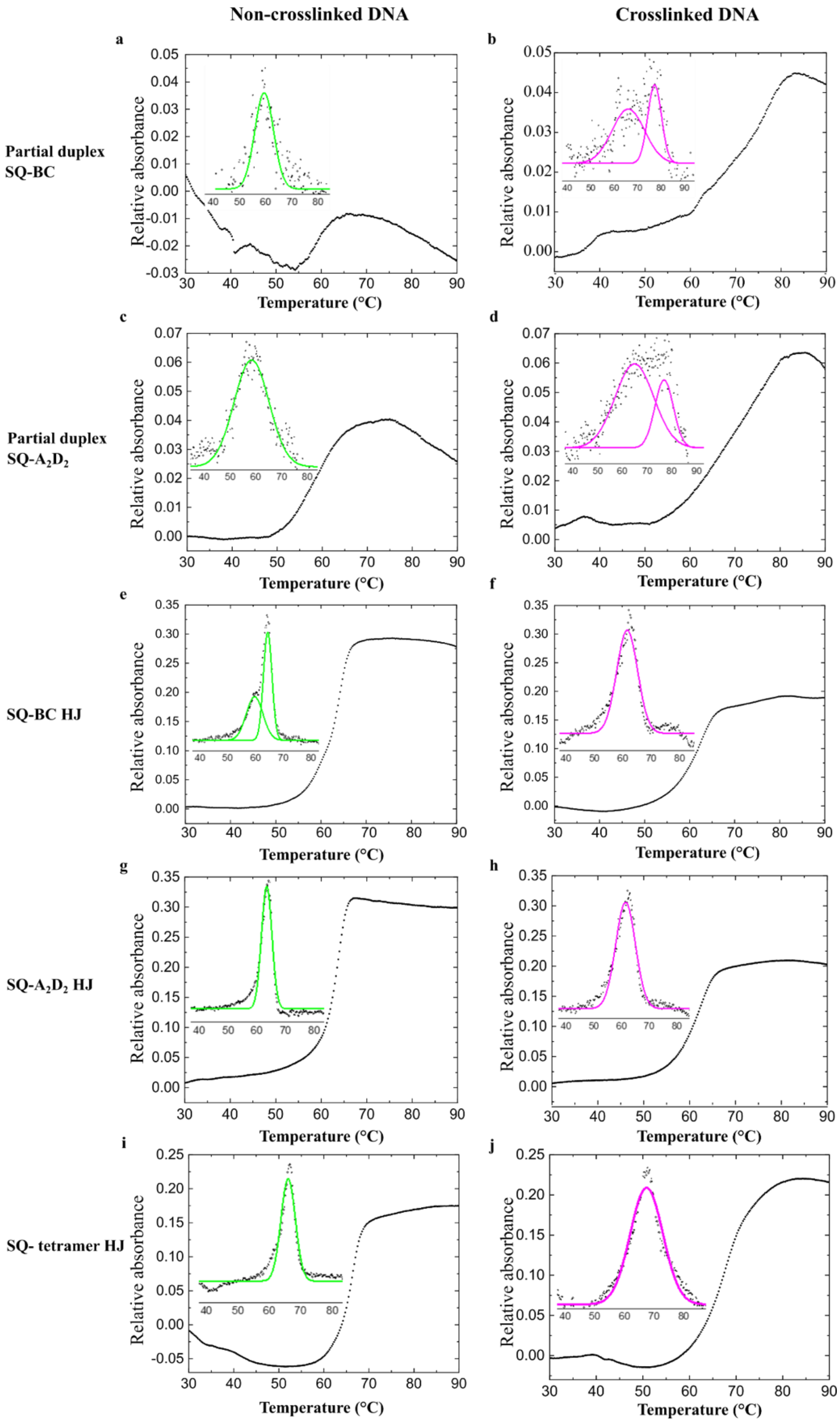

Figure S10. Melting profiles of crosslinked and non-crosslinked DNA in 1× TBE, 15 mM $MgCl_2$. The sample concentration was 1.5 μM. The thermal denaturation was monitored via absorption at 260 nm. The inserts show the first derivative as a function of temperature and fitted Gaussian curves.

Table S5. Melting temperature of crosslinked and non-crosslinked DNA in 1× TBE, 15 mM $MgCl_2$.

Non-Crosslinked DNA Construct	Melting temperature (T_m), °C	Crosslinked DNA Construct	Melting temperature (T_m), °C
Partial duplex SQ-BC	59.3	Partial duplex SQ-BC$^{<>}$	65.9; 77.1
Partial duplex SQ-A$_2$D$_2$	58.2	Partial duplex SQ-A$_2$D$_2^{<>}$	64.7; 76.7
SQ-BC	59.6; 64.3	[a]SQ-BC$^{<>}$ HJ	61.4
SQ-A$_2$D$_2$	63.4	[b]SQ-A$_2$D$_2^{<>}$ HJ	61.3
SQ-tetramer HJ	66.1	[c]SQ-tetramer$^{<>}$ HJ	67.4
Unmodified HJ	61.5		

[a]Crosslinked SQ-BC$^{<>}$ HJ was obtained by combining a purified crosslinked partial duplex SQ-BC$^{<>}$ with unmodified single strands A and D in equimolar amounts followed by annealing for 4 min at 94 °C and cooling to room temperature. [b]Crosslinked SQ-A$_2$D$_2^{<>}$ HJ was obtained by combining a purified crosslinked partial duplex SQ-A$_2$D$_2^{<>}$ with unmodified single strands B and C in equimolar amounts followed by annealing for 4 min at 94 °C and cooling to room temperature. [c]SQ-tetramer templated by doubly crosslinked HJ.

Supporting Information 8: *Spectral properties of SQ-labeled DNA constructs*

The absorption spectra of crosslinked and non-crosslinked constructs were recorded in 1× TBE with 15 mM $MgCl_2$ (**Figure S11**).

The absorption spectrum of monomer **SQ-A** exhibited a strong low energy absorption band at 645 nm with a weak vibrionic shoulder.. Most non-crosslinked and crosslinked SQ-DNA aggregates exhibited a blue shift of their absorption maxima compared to the monomer (**Table S6**). The exception was the crosslinked **SQ-A$_2$D$_2^x$** HJ with the 2 nm red-shift with respect to the monomer.

The circular dichroism (CD) spectra were collected for the non-crosslinked SQ-BC dimer, crosslinked partial duplex SQ-BC, and non-crosslinked and doubly crosslinked SQ-tetramers. The CD spectra were collected on JACSO J-1500 spectrometer. The samples (100 μL) were placed directly in a 1 cm path length micro cuvette (Jasco). CD spectra were monitored in a 230-800 nm of wavelength range at a speed of 200 nm/min.

All four constructs showed a strong couplet at the 260-280 nm region representing a well-formed duplex DNA. Non-crosslink tetramer showed a strong signal in the visible area of the CD spectra while crosslinked double stranded BC dimer or non-crosslinked BC HJ aggregates did not exhibit any signal in the visible area of the CD spectra.

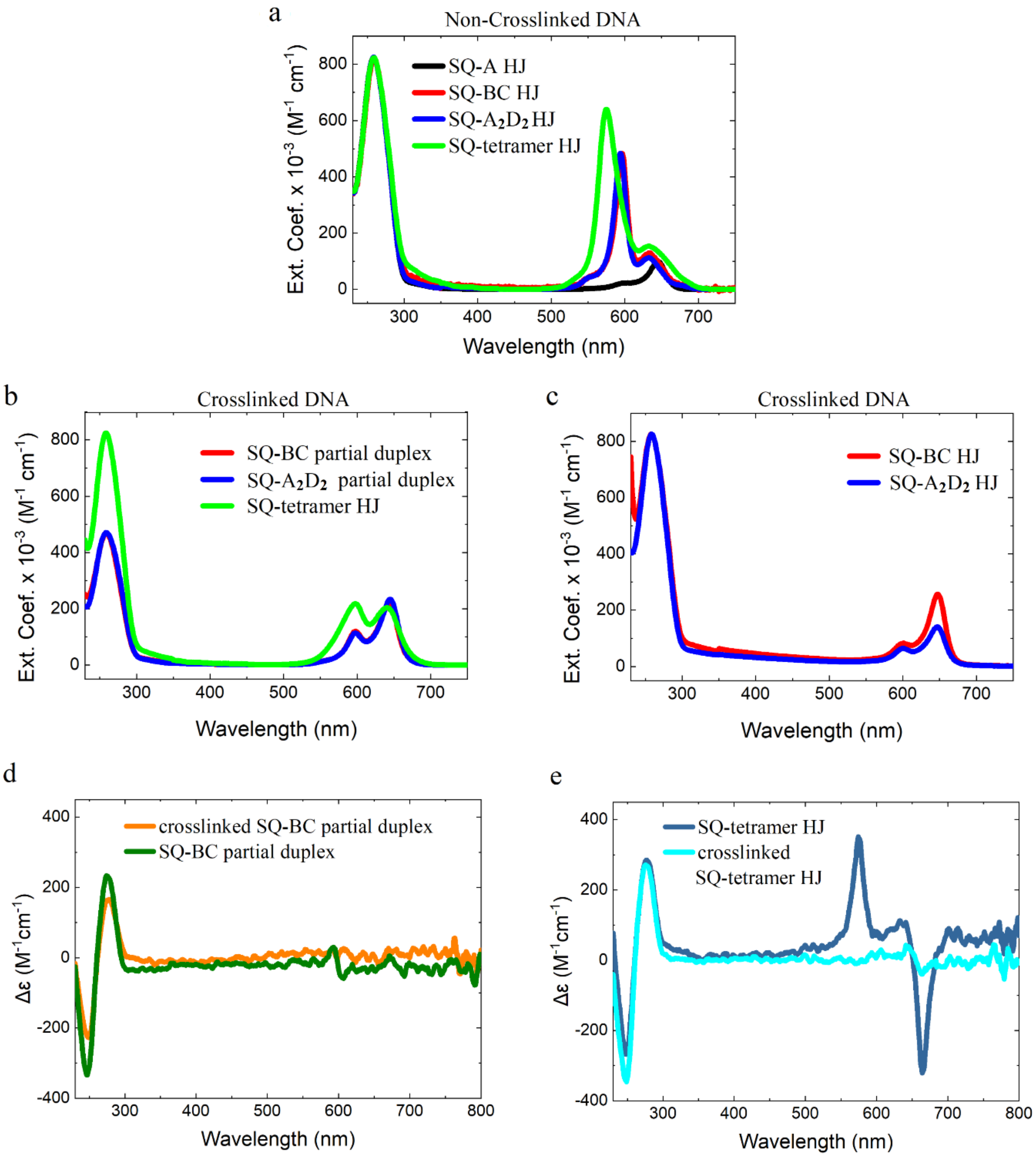

Figure S11. (**a, b, c**) Acquired steady-state absorption spectra converted to extinction of squaraine-DNA constructs in 1× TBE, 15 mM $MgCl_2$ at room temperature. (**d, e**) Full CD spectra of the DNA-Squaraine dye constructs recorded in 1× TBE, 15 mM $MgCl_2$ at room temperature. The DNA-dye construct concentration was 1.5 μM.

Table S6. Absorbance of crosslinked and non-crosslinked SQ-labeled DNA in 1× TBE, 15 mM $MgCl_2$.

Non-Crosslinked DNA Construct	Absorption (nm)	Crosslinked DNA Construct	Absorption (nm)
SQ-monomer	645		
Partial duplex SQ-BC	-	Partial duplex SQ-BC$^{<>}$	598; 644
Partial duplex SQ-A_2D_2	-	Partial duplex SQ-$A_2D_2^{<>}$	598; 645
SQ-BC	596; 633	[a]SQ-BC$^{<>}$ HJ	[a]601; 648
SQ-A_2D_2	594; 632	[b]SQ-$A_2D_2^{<>}$ HJ	[b]600; 647
SQ-tetramer HJ	575; 633	[c]SQ-tetramer$^{<>}$ HJ	598; 641

[a]Crosslinked SQ-BC$^{<>}$ HJ was obtained by combining a purified crosslinked partial duplex SQ-BC$^{<>}$ with unmodified single strands A and D in equimolar amounts followed by annealing for 4 min at 94 °C and cooling to room temperature. [b]Crosslinked SQ-$A_2D_2^{<>}$ HJ was obtained by combining a purified crosslinked partial duplex SQ-$A_2D_2^{<>}$ with unmodified single strands B and C in equimolar amounts followed by annealing for 4 min at 94 °C and cooling to room temperature. [c]SQ-tetramer templated by doubly crosslinked HJ.

Supporting Information 9: *References*

1. Schneider, C. A.; Rasband, W. S.; Eliceiri, K. W. Nih Image to Imagej: 25 Years of Image Analysis. *Nature Methods* **2012,** *9*, 671-675.
